# The Influence of the Functional Group on the Physicochemical and Biological Properties of New Phenanthro[9,10-d]-Imidazole Derivatives

**DOI:** 10.3390/molecules29194703

**Published:** 2024-10-04

**Authors:** Slawomir Kula, Paweł Kalarus, Łukasz Kaźmierski, Anna Biernasiuk, Przemysław Krawczyk

**Affiliations:** 1 Institute of Chemistry, Faculty of Science and Technology, University of Silesia, Szkolna 9 St., 40-007 Katowice, Poland; pawelkalarus2000@gmail.com; 2 Department of Oncology, Radiotherapy and Oncological, Faculty of Medicine, Collegium Medicum, Nicolaus Copernicus University, M. Curie Skłodowskiej 9, 85-094 Bydgoszcz, Poland; lukasz.kazmierski@cm.umk.pl; 3 Department of Pharmaceutical Microbiology, Faculty of Pharmacy, Medical University of Lublin, 20-093 Lublin, Poland; anna.biernasiuk@umlub.pl; 4 Department of Physical Chemistry, Faculty of Pharmacy, Collegium Medicum, Nicolaus Copernicus University, Kurpińskiego 5, 85-950 Bydgoszcz, Poland; przemekk@cm.umk.pl

**Keywords:** phenanthroimidazole derivatives, phenanthro[9,10-d]imidazole derivatives, rhodanine-3-acetic acid derivatives, fluorescent dyes, bioimaging, antimicrobial and antifungal activity

## Abstract

The search for safe, cheap, and repeatable diagnostic methods is a fundamental research goal. Currently, great hope is placed on fluorescence imaging. However, the development of this method mainly depends on efficient fluorescent probes. Designing and obtaining new probes with potential applications in fluorescence imaging is very difficult because compounds of this type must meet several requirements related to their properties. Therefore, this article attempted to obtain and study new phenanthro[9,10-d]-imidazole derivatives (PK1–PK3) with potential application as fluorescent probes for fluorescence imaging. The main goal of the work was to assess the effect of two functional groups (such as the formyl group (PK2) and rhodanine-3-acetic acid (PK3)) on selected physicochemical properties and possibilities of practical application of the considered compounds. The conducted studies proved that the influence of the functional group is significant, as it causes a bathochromic shift in both absorption and emission results (by the order PK1 < PK2 < PK3). Moreover, all compounds could stain live cells cultured in vitro. The staining efficiency was not affected by the cell line, thanks to which we obtained the correct staining of both mouse and human cell lines. PK3 was the most attractive of the tested compounds due to its staining potential of live cells and retention after fixation. Our results also showed some antibacterial and antifungal activity of the newly synthesized compounds (PK1–PK3). Among them, PK3 showed the highest antimicrobial effect, especially against Gram-positive bacteria.

## 1. Introduction

Stable, highly reproducible diagnostic tools are essential for sensitive, rapid, and accurate verification of disease lesions. Biological imaging is a range of methods that allow for the detection, visualization, and characterization of biological structures [1,2,3,4,5]. Typical biological imaging methods include well-known diagnostic techniques such as X-ray, magnetic resonance imaging, computed tomography, single-photon emission tomography, and fluorescence imaging [6,7,8,9,10,11]. These techniques are widely used in medical research due to their extraordinary ability to visualize essential details without significant interference in life processes [12]. Moreover, depending on the chosen diagnostic method, we can visualize subcellular, cellular, and multicellular structures, tissues, and organs. An additional advantage of the mentioned techniques is the ability to provide diagnostic information in real time. However, each imaging method has different sensitivity, resolution, and limitations. The perfect solution would be combining several imaging techniques to obtain multiple capabilities from many methods, but this is impossible. This is only possible for methods with similar diagnostic parameters to intensify a specific method’s feature [6,13]. Among various imaging techniques, fluorescence imaging is particularly distinguished [2,5,12,14]. This is one of the most popular methods of observing cells, allowing them to be examined without toxic and time-consuming staining processes. Its main advantages are that it provides information in real time, is non-invasive, and has high sensitivity and resolution [15]. However, fluorescence imaging requires fluorescent probes, and this method’s further development depends on them [6]. Therefore, the primary goal of many researchers is not to improve imaging methods but to obtain contrast agents that will solve the problem of imperfect methods [6,13]. Fluorescent dyes must meet several requirements for use. Chemical compounds designed for this purpose typically have a planar, symmetric molecular architecture that favors the absorption of near-infrared light [5,12,14,16,17,18]. Typically, derivatives with delocalized π-type bonds are used [5,12,14,17]. Dye molecules should bind specifically to biomolecules and emit fluorescent signals [2,19,20,21]. Such a signal should be characterized by appropriate intensity and result from excitation with radiation of a proper wavelength [15,19,20]. The Stokes shift [15,16,17,19] is also essential because an inappropriate selection of wavelength will not only not ensure a good penetration depth of living tissues but may also lead to their damage [16]. The best solution may seem to be to use a probe with the highest possible fluorescence intensity (due to the high signal-to-noise related) [15,19]. This is not always the right solution because it may affect other characteristics of the dye, such as its toxicity [22,23]. Moreover, it is crucial that such a chemical compound has a group in its structure that allows for easy binding to the biomolecule [2,14,16,22,24]. Examples of such groups are aldehyde, thiol, or amino groups [14,16]. The solubility of the dye in solvents that are not harmful to the living organism is also crucial [14,15]. The least harmful solution is to use water as a solvent [14,18], but dyes are not always soluble in it. Other solvents that can be used medically are ethyl alcohol or dimethyl sulfoxide. The comprehensiveness and interdisciplinary nature of the research that must be carried out to select new fluorescent dyes for use in fluorescence imaging makes it a difficult task.

Considering the above, this work attempted to obtain and investigate new phenanthro[9,10-d]-imidazole derivatives with potential use as fluorescent probes for fluorescence imaging. This article continues our previous research on fluorescent probes based on the phenanthro[9,10-d]-imidazole core [5,12,14,17,25,26,27]. The primary aim of the work was to assess the influence of two functional groups (such as the formyl group and rhodanine-3-acetic acid) on selected physicochemical properties and the possibilities of practical application of the considered chemical compounds. Moreover, all obtained phenanthro[9,10-d]-imidazole derivatives had an aromatic substituent (in the form of a phenyl ring) located at the nitrogen atom in the central imidazole ring. The presented research results were enriched with DFT calculations, allowing a better understanding of the experiments performed.

## 2. Results and Discussion

### 2.1. Synthesis

**Figure 1:**
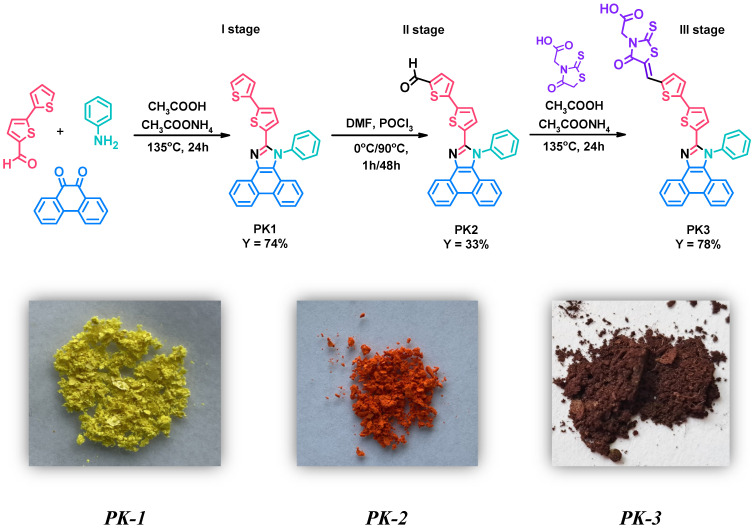
Method for the synthesis of PK1–PK3 derivatives along with photos of the obtained solids.

Research in this work began with the synthesis of 2-(2,2′-bithiophen-5-yl)-1-phenyl-1H-phenanthro[9,10-d]-imidazole (PK1)—first stage. This compound was obtained in the Debus–Radziszewski reaction between 2,2′-bithiophene-5-carboxaldehyde, aniline, phenanthrenequinone, and ammonium acetate (Figure 1—I stage). Acetic acid was used as the reaction medium. For this type of reaction, heating the reaction mixture at reflux temperature has proven highly advantageous. The crude product was precipitated, filtered, and washed thoroughly with distilled water. Finally, PK1 was purified by column chromatography. The yield of the reaction was 74%. The detailed procedure for synthesizing PK1 was also described in our previous studies aimed at searching for new emitters for OLEDs [28]. Then, in the second stage, the obtained 2-(2,2′-bithiophen-5-yl)-1-phenyl-1H-phenanthro[9,10-d]-imidazole (PK1) was subjected to a formylation reaction using phosphorus(V) oxychloride (POCl3) and N,N-dimethylformamide (DMF) (Figure 1—II stage). POCl3 was added slowly (drops) to the reaction mixture at a temperature of 0 °C. After one hour, the temperature of the reaction mixture was gradually raised to 90 °C. This temperature was then maintained for two days. Interestingly, DMF acted as a solvent and reagent in this reaction. After heating, the crude product was precipitated, filtered, washed thoroughly with distilled water and purified by column chromatography. Thanks to the procedure used, 5′-(1-phenyl-1H-phenanthro[9,10-d]imidazol-2-yl)-2,2′-bithiophene-5-carbaldehyde (PK2) was obtained with a yield of 33%. A significant advantage of the presented method compared to coupling reactions used to synthesize structurally similar compounds is its simplicity and lower price due to the lack of an expensive catalytic system [29]. The third step consisted of the Knoevenagel condensation of the previously obtained PK2 with rhodanine-3-acetic acid in the presence of acetic acid as a solvent (Figure 1—III stage). This reaction was carried out at the boiling temperature of the reaction mixture. After completing the synthesis of 2-[5-((5′-(1-phenyl-1H-phenanthro[9,10-d]imidazol-2-yl)-2,2′-bithiophene-5-yl)methylene)-4-oxo-2-thioxo-thiazolidin-3-yl]acetic acid (PK3), it was filtered, washed thoroughly with distilled water, and dried. Interestingly, PK3 did not require additional purification methods. The yield of the reaction was 78%. The structure of the obtained compounds (PK1–PK3) was confirmed by NMR spectroscopy (^1^H and ^13^C). The necessary NMR spectra can be seen in the SI (Figures S1–S6).

### 2.2. Thermal Characteristics

Phenanthro[9,10-d]imidazole derivatives are generally characterized by excellent thermal properties [5,14,27,28,30]. By analyzing the PK1–PK3 compounds in terms of thermal properties, we can successfully notice the influence of functional groups on selected parameters. In the case of the melting temperature (Tm) determined using DSC (differential scanning calorimetry), the introduction of an aldehyde group into the structure of the PK1 derivative does not increase Tm (Table 1). PK2 has a melting point only 2 °C higher than PK1. In turn, the PK3 derivative containing a heterocyclic rhodanine-3-acetic acid ring in its structure shows a significant increase in the melting point compared to PK2 (by 88 °C) and PK1 (by 90 °C). The comparison of the discussed compounds in terms of their thermal stability determined using TGA (thermogravimetric analysis) looks completely different. Importantly, all derivatives show a 5% weight loss above 300 °C, which proves their high stability (Table 1). However, despite the difference in structure between PK2 and PK3, we do not observe a change in T5. Both derivatives only have higher stability by approximately 18–19 °C than PK1. We can see a similar effect considering the mass loss parameter of 10% but with a smaller temperature difference than in the case of PK1. A graphical illustration of the thermal tests (DSC and TGA) is provided in the SI (Figures S7–S10).

### 2.3. Optical Properties

To better assess the influence of functional groups on the properties of PK1–PK3 derivatives, they were subjected to optical tests. Experiments were performed in five solvents of different polarities. The polarity of solvents was assessed based on their dielectric constant (ε) values. It is known that the higher the dielectric constant of a solvent, which measures its polar properties, the more easily polar molecules or ionic crystals dissolve in it. Solvents composed of molecules with weak polar properties (low dielectric constant) are, on the other hand, suitable solvents for nonpolar molecules. The measurements were carried out on solutions with a concentration of 2.5 × 10^−5^ mol·dm^−3^ because they give an absorbance below one in the full spectrum range, which allows for the exclusion of a possible aggregation problem. All data, photoluminescence and absorption spectra for studied compounds in different media are collected in Table 2 and Figures S11–S14 in Supplementary Materials.

**Figure 2:**
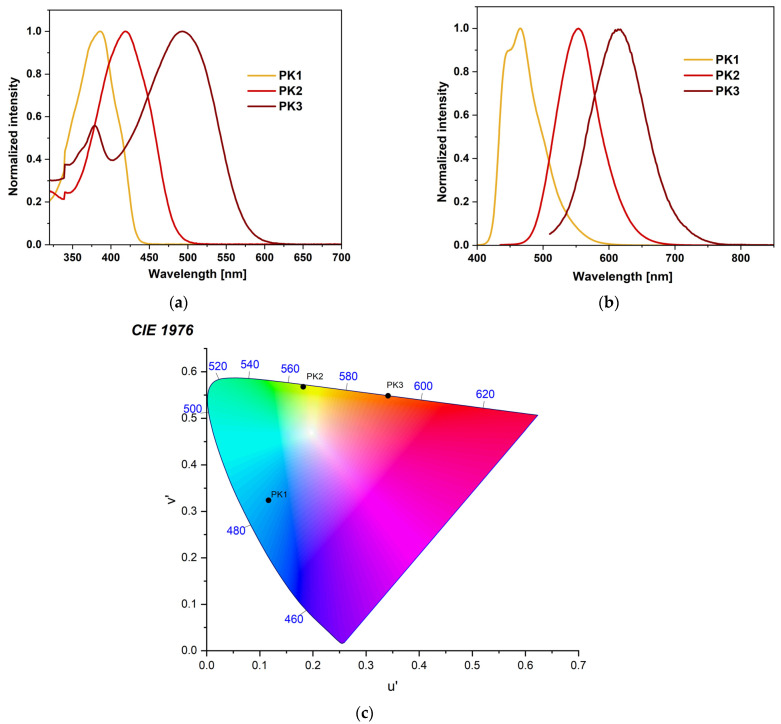
Normalized (**a**) UV–Vis, (**b**) emission spectra, and (**c**) chromaticity plot of PK series in DMSO.

The absorption profile for each compound differs significantly. The absorption peak for PK1 is narrow and lies in the range with a maximum of 372–386 nm (Figure 2a). It can be attributed to the π→π* transition associated with the phenanthro[9,10-*d*]-imidazole [28,30,31,32,33,34,35]. For PK2, this peak is slightly broader and milder (Figure 2a). It is located in the range with a maximum between 406–421 nm. It is most likely a mixture of the π→π* transition and intramolecular charge transfer (ICT) [5,14,27,35]. Comparing the PK2 compound with the structurally similar IA-2, it can be concluded that the spectrum’s shape does not change [5]. The maxima of PK2 peaks are slightly blue-shifted. The phenyl substituent attached to the N1 position is responsible for this. PK3 has two absorption peaks. The first of them has a scope similar to PK1. The second one is quite wide, between 581–614 nm, which may result from intramolecular charge transfer (ICT), characteristic of rhodanine-3-acetic acid derivatives [36,37,38]. Moreover, the effect of solvent polarity (from nonpolar to polar, according to the increasing dielectric constant value) on the absorption behavior is minimal for all compounds (Figure S11). As observed, the modification of the 2,2′-bithiophene substituent causes a significant bathochromic shift consistent with the order PK1 < PK2 < PK3 (Figure 2a). This can be attributed to an electron-withdrawing aldehyde group (-CHO) in PK2 and an electron-withdrawing heterocyclic rhodanine-3-acetic acid ring in PK3 [5,36]. Interestingly, we observe lower absorption maxima in MeOH and MeCN than in toluene. This phenomenon can be attributed to the hydrogen bonding interaction of the solvent molecules with the imidazole ring. This typical behavior can be ascribed to the hydrogen-bonding interactions, which probably retard the reorientation of the fluorophore by hindering the rotation of the aryl unit [36,39]. Then, all three compounds were tested for their stability in DMSO solution. This is a crucial parameter from the point of view of bioimaging. The results indicated that the profile of absorption spectra did not change during 24 h measurements (Figure S12). This shows the excellent stability of these compounds. The next stage of the research was photoluminescence testing in solution. PK1 shows two emission bands in the range of 440–465 nm. There is no solvent dependence for the derivative in question. The spectra in all presented solvents overlap and the Stokes shift is tiny (Figure S11). The PK1 bands are well-organized fluorescence bands that suggest emission from the locally excited singlet (^1^LE) emission state [27,36]. Moreover, these spectra have a similar shape to analogous compounds described in the literature [28,30]. The emission band of PK2 is in the range of 495–564 nm. It can be seen that the effect of the medium polarity (change in the dielectric constant value) on the emission behavior is more pronounced than the influence on the absorption behavior. This proves polar solvents’ better stabilization of the singlet excited state [5,14,17]. The greater the polarity of the solvent, the more significant the red shift of the band and the greater the Stokes shift (Figure S11). This bathochromic shift was assigned to ICT from an electron-rich phenanthro[9,10-d]imidazole residue to an electron-deficient aldehyde group [5,14]. The emission band of PK3 is in the range of 571–614 nm. It is also an ICT band. In the case of PK3, we observe a less pronounced solvatochromic effect than in the case of PK2. Interestingly, the spectrum of the PK3 molecule in MeOH is significantly blue-shifted (Figure S11). Moreover, a less pronounced shoulder peak at 571 nm is observed in the MeCN spectrum. This may result from a more complex molecule with more functional groups. This increases the possibility of creating a more rigid solvation cage due to hydrogen bonds with solvents. Therefore, a molecule can experience two different local environments in the ground state itself [36]. Similar phenomena were observed for structurally similar compounds [36]. Comparing the derivatives with each other, it is noticeable that the modification of the C2 substituent causes a red shift of 88 nm and 149 nm in DMSO (Figure 2b). Moreover, emission chromaticity diagrams were determined for the given molecules (Figure 2c and Table S3). Analyzing the chromaticity diagram and emission spectra, it can be concluded that small structural modifications significantly affect the emission spectra. PK1 had the highest quantum yield (26.8%) with blue-shifted emission and lower Stokes shifts (Table 2) compared to PK2 and PK3. For PK2 the quantum yield was 14.5% and for PK3 only 0.6%. A typical phenomenon of the pure nature of ICT emissions is the decrease in QY [38].

Dipole moments were determined for all molecules using the shift between the absorption and emission maxima Δ*v* = (*v*_abs_ − *v*_em_) as a function of solvent polarity. The Lippert–Mataga equation was used [36,40]:

$$v_{abs}-v_{em}=\Delta v=\frac{2}{hca^{3}}{\left(\mu_{e}-\mu_{g}\right)}^{2}\Delta f+Const.$$

where Δ*v* is the Stokes shift, *μ*_g_ and *μ*_e_ are the dipole moments in the ground and excited states, *c* is the speed of light, *h* is Planck’s constant, *a* is the radius of the Onsager cavity, and Δ*f* is determined from the formula. The orientation polarizability (Δ*f*) of the solvent is determined using the following equation:

$$\Delta f=\frac{\varepsilon -1}{2\varepsilon +1}-\frac{n^{2}-1}{2n^{2}+1}$$

where ε is the dielectric constant of the solvent and n is the optical refractive index of the solvent. A plot of Δ*v* versus Δ*f* gives Δ*μ* from the slope. The value of *a* was calculated theoretically and is 6.09 Å, 5.98 Å, and 6.66 Å for PK1, PK2, and PK3, respectively. The dipole moment values for PK1 and PK3 were similar and were 10.77 D and 10.75 D. PK2 has the largest dipole moment of 13.57 D. A graphical representation of the Lippert–Mataga plots is provided in the Supplementary Materials (Figure S14).

### 2.4. DFT Calculations

#### 2.4.1. Chemical-Optical Properties

For all evaluated derivatives, the HOMO→LUMO transition is inherently linked to charge-transfer (CT) excitation. In each instance, HOMO electrons are delocalized across the entire molecular structure, with the exception of the region above the benzene ring attached to the imidazole unit and the –COOH group in PK3 (Figure S15). Conversely, LUMO electrons are redistributed away from the phenanthroimidazole moiety towards the bithiophene unit. Notably, in the case of PK3, electron accumulation is absent in the -CH2–COOH group, while an increase in electron density is observed in the thioxothiazolidin ring region. As indicated in Table S1, for the PK1 molecule, the energy gap between the HOMO and LUMO orbitals (ΔEGAP) widens with increasing medium polarity and ${\Delta \Delta E}_{GAP}^{DMSO-Tol}$ is 0.0022 eV. Conversely, for the remaining derivatives, the ΔEGAP value decreases, with PK2 ${\Delta \Delta E}_{GAP}^{Tol-DMSO}$ exhibiting 0.0046 eV and PK3 showing 0.0321 eV. Notably, PK3 also displays the lowest ΔEGAP values overall. The introduction of the electron-withdrawing substituent –COH into the PK1 molecule further reduces this value by an average of 0.54 eV. As illustrated in Table S1, chemical hardness (η) diminishes with increasing solvent polarity, while softness (σ) correspondingly increases—except in the case of PK1, where a slight decrease in softness is observed. Among the derivatives, PK1 is characterized by the highest η values, indicating superior stability relative to the others. In contrast, the lowest η value observed for PK3 suggests it possesses the highest chemical reactivity, thereby facilitating charge transfer. This observation is confirmed by the values of σ, where $\sigma_{PK3}>\sigma_{PK2}>\sigma_{PK1}$. Negative chemical potential (μ) values signify that the absorption process is thermodynamically spontaneous. Concurrently, the elevated μ values for PK3 suggest a greater propensity for electron detachment from the equilibrium system. The global electrophilicity (ω) values further establish PK3 as the most electrophilic species among the examined systems. Additionally, the high electronegativity observed implies a facile formation of coordination bonds during diverse chemical processes.

To identify potential regions for electrophilic (negative: red and yellow) and nucleophilic (positive: blue) attacks in the tested derivatives, molecular electrostatic potential (MEP) surfaces were computed (Figure S16). In the case of PK1, the most negative regions are primarily located within the phenanthroimidazole moiety, particularly around the imidazole nitrogen atom that is not bonded to the benzene ring. The attachment of the –COH group to the outer thiophene ring weakens this electrophilic zone, causing a shift of the negative potential toward the oxygen atom of the aldehyde group. For PK3, the maximum negative potential is situated on the oxygen atom of the carboxyl group, indicating that the 2-(4-oxo-2-thioxothiazolidin-3-yl)acetic acid moiety is more prone to electrophilic attack compared to the phenanthroimidazole segment. In contrast, the PK1 and PK2 molecules exhibit lower susceptibility to nucleophilic attack than PK3, where the highest concentration of positive charge is localized on the hydrogen atom of the –COOH group. The MEP analysis suggests that specific solvent–solute interactions are likely to occur for the tested derivatives, which may, in turn, influence the positioning of the maxima in the absorption and fluorescence spectra.

The vertical values of the absorption band maxima ($\lambda_{ABS}$) presented in Table S2 are in good agreement with the experimental measurements, although the TDDFT method slightly underestimates the excitation energy values. The average deviation ${\Delta \lambda }_{ABS}^{TDDFT-Exp}$ is 8.72 nm, 7.04 nm, and 13.32 nm for PK1, PK2, and PK3, respectively. The largest discrepancies are observed in MeOH and not taking this solvent into account reduces the error value to 5.56 nm, 4.18 nm, and 7.51 nm. In turn, the values determined within the cLR ($\lambda_{cLR}$, Table S2) approximation are much more overestimated, and the error increases to 12.96 nm, 15.10 nm, and 17.85 nm. Moreover, the cLR method shifts the maximum of the absorption bands towards wavelengths longer than TDDFT by an average of 4.23 nm, 8.06 nm, and 6.94 nm for PK1, PK2, and PK3, respectively. Attaching an aldehyde group to the PK1 derivative causes a bathochromic effect by an average of 32.92 nm. Expansion of the structure with another aromatic ring shifts $\lambda_{ABS}$ further towards longer wavelengths and the average ${\Delta \lambda }_{ABS}$ for PK2→PK3 is 75.67 nm and 108.60 nm for PK1→PK3. The tested derivatives are characterized by non-monotonic behavior of the position of the absorption band maxima. For both vertical and measured $\lambda_{ABS}$, the Tol→CHCl_3_→MeOH transition is accompanied by a hypsochromic shift, and MeOH→MeCM→DMSO by a bathochromic effect. Moreover, for the transition between solvents with limiting dielectric constant values (Tol→DMSO), the theoretical maximum $\lambda_{ABS}$ shifts towards shorter wavelengths by 1.34 nm and 1.25 nm, respectively. In turn, a bathochromic shift of 4.63 nm is observed for PK3. In the case of experimental measurements, the hypsochromic effect occurs only for PK2 (3 nm), while for the others there is a shift towards longer wavelengths (2 nm and 1 nm).

The non-monotonic behavior of the $\lambda_{ABS}$ position is consistent with the MEP analysis and can be attributed to the enhanced stabilization and increased polarization of the ground state in polar environments, leading to higher excitation energies. However, the polarity of the excited state (${\Delta \mu }_{CT-GS}$) follows a reverse trend (Table S3). For all molecules, the μGS values are lower than the dipole moment of the lowest-lying excited state, which is indicative of positive solvatochromism. Furthermore, both μCT and ${\Delta \mu }_{CT-GS}$ increase with the polarity of the medium. PK3 is characterized by the strongest excited-state polarity, with the ${\Delta \mu }_{CT-GS}$ value being twice that of PK1. These observations suggest the presence of short-range specific interactions, such as self-aggregation and hydrogen bonding. Nonetheless, purely electrostatic contributions to the PK(1,2,3)–solvent interactions are unlikely.

**Figure 3:**
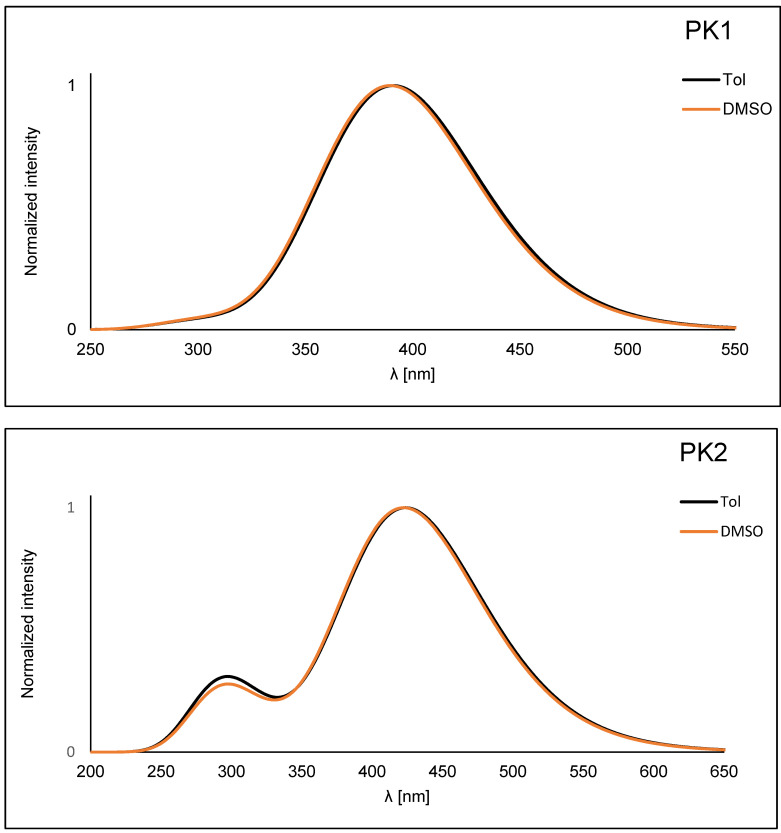

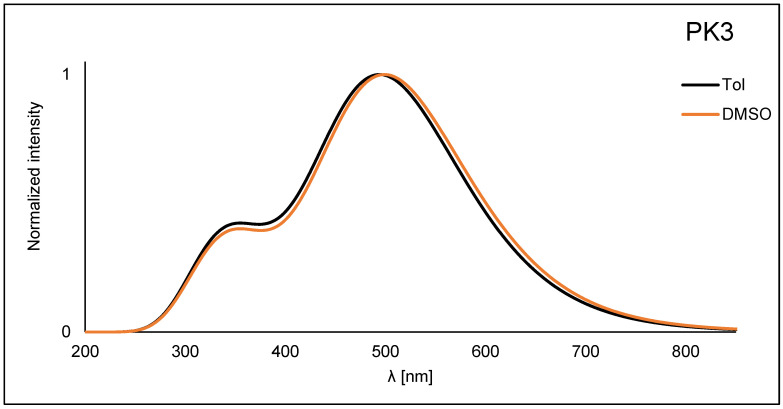
The theoretically determined vertical excitation energies.

A graphical representation of the theoretical absorption bands (Figure 3), similarly to those recorded experimentally, indicates that for PK2 and PK3 additional bands appear hypsochromically shifted relative to the main band, at 297.40 nm and 355.00 nm, respectively. In the case of PK1, it appears at 315.13 nm, but its low intensity does not reveal a characteristic peak, but only an irregularity in the spectrogram. For both solvents, they occur at the same wavelength, but in toluene they are slightly more intense. Therefore, in addition to the HOMO→LUMO transition, contributions from other orbitals should also be expected. For this purpose, an analysis of density differences was performed. Designated difference in total density computed for the ground and excited states (Δρ(r), Figure S17) suggests that the location of the depletion sites (blue) and the density increment zones (purple) do not depend significantly on the expansion of the molecular structure. For each derivative, the depletion zones extend throughout the entire molecule. In turn, the increment sites accumulate on bithiophene and the substituents attached to it that distinguish the molecules. The specificity of the PK3 system means that these zones do not appear on the –COOH group, while they are very visible on the –COH group. However, both zones are not visible within the benzene ring.

The Table S4 shows the DCT values as a measure of the length of the electron transfer associated to an electronic transition. The index, based only on the computed electronic density for the ground and excited states, quantifies the charge-transfer (CT) length as the distance between the barycenters of the density increment and depletion regions upon electronic excitation. This index shows a monotonous increase for the Tol→MeOH transition and then a decrease in value for the MeOH→DMSO transition. In the first case, the ${\Delta D}_{CT}^{MeOH-Tol}$ values are higher and are 0.117 Å, 0.713 Å, and 0.747 Å for PK1, PK2, and PK3, respectively. In turn, the ${\Delta D}_{CT}^{MeOH-DMSO}$ values do not exceed 0.050 Å and are 0.036 Å, 0.009 Å, and 0.043 Å. Analogously to the previous dependencies, the highest of the length of the electron transfer are described by the PK3 derivative, and ${\Delta D}_{CT}^{PK3-PK1}$ and ${\Delta D}_{CT}^{PK3-PK2}$ are 3.468 Å and 1.094 Å, respectively. The DCT index confirms the CT character of the discussed derivatives and the contributions from HOMO→LUMO transition. For all molecules, the amount of transferred charge decreases monotonously as a function of environmental polarity, and the ${\Delta q}_{CT}^{Tol-DMSO}$ values are 0.026 e, 0.019 e, and 0.009 e for PK1, PK2, and PK3. In terms of the average value (AV) of this quantity, the tested compounds are arranged in the following series: $PK3\left(q_{CT}^{AV}=6.505e\right)>PK2\left(q_{CT}^{AV}=5.411e\right)>PK1\left(q_{CT}^{AV}=3.037e\right)$. As previously mentioned, contributions from other orbitals are not negligible. Therefore, an additional maximum shifted towards shorter wavelengths appears. For PK1, this band is associated with the HOMO→LUMO+3 (64%), HOMO→LUMO+2 (20%), and HOMO-1→LUMO (9%), for PK2 with HOMO-2→LUMO (78%), HOMO-6→LUMO+1 (11%), and HOMO-6→LUMO (3%), while for PK3 with HOMO→LUMO+1 (90%), HOMO-1→LUMO (5%), and HOMO-2→LUMO (2%) transitions. This indicates that pure electrostatic contributions to the solute–solvent interactions should not occur and short-range specific interactions may be present.

Similarly to the $\lambda_{ABS}$, in the presence of polar solvents non-monotonic behavior in the position of $\lambda_{FL}$ is observed (Table S2). This proves that the tested markers are characterized by a charge-separated ground state and a neutral excited state. The maximum emission spectrum for PK3 is most bathochromically shifted, while for PK1 it is most hypsochromically shifted. PK2 is characterized by the highest values of the Stokes’ shift (${\Delta \nu }^{St}$), and in terms of this quantity the molecules are arranged in the following series: $\Delta \nu_{PK2}^{St}\left(AV=119.05\;nm\right)>\Delta \nu_{PK3}^{St}\left(AV=105.98\;nm\right)>\Delta \nu_{PK1}^{St}\left(AV=76.34\;nm\right)$. Therefore, from the point of view of bioimaging applications, PK2 and PK3 can be used as valuable fluorescent probes.

Theoretically calculated NLO values are shown in Table S5. The values of $\langle \alpha \rangle$ and β increase monotonously as a function of the solvent polarity. Molecule PK3 is characterized by the highest non-linear response, while PK1 by the lowest. The $\langle \alpha \rangle$ values for PK3 are higher on average by 365.82 a.u. compared to PK1 and by 299.15 a.u. relative to PK2. In case β, these differences are much greater. The average β value for PK1 is 1968.25 a.u., and the introduction of the –COH group to the system increases this value by over eight times, to the level of 16,885.67 a.u. Expanding the structure with another aromatic system results in a further increase in β to the value of 53,514.56 a.u., which makes it more than three times higher compared to PK2 and more than 27 times higher than PK1. This indicates that the presence of the 2-(4-oxo-2-thioxothiazolidin-3yl)acetic acid not only shifts the maximum of the absorption bands towards longer wavelengths, but also significantly maximizes the values of dipole moments and increases the nonlinear response of the system.

Table S6 shows the two-photon absorption cross-section (TPA) values based on the CAM-B3LYP functional. Similarly to β, in terms of these quantities, the tested molecules are arranged in the following order: PK3>PK2>PK3. The first derivative is described with an average $\langle \delta^{OF,AV}\rangle$ value of 3132.73 a.u. and $\sigma_{OF}^{(2)AV}$ of 10.79 GM. Such low TPA values make this molecule unsuitable for applications in nonlinear optics. The introduction of an aldehyde group into the system increases the average value of $\langle \delta^{OF,AV}\rangle$ by 8.6 times (27,088.43 a.u.) and $\sigma_{OF}^{(2)AV}$ by seven times (75.84 GM). Replacing the –COH substituent with the 2-(4-oxo-2-thioxothiazolidin-3yl)acetic acid leads to a further increase in these values to the level of 77 a.u. and 99 GM. On this basis, the PK2 and PK3 derivatives should be considered as valuable probes in two-photon imaging and as visual tools in real-time dynamic imaging in in vivo and in vitro research.

The tested derivatives are characterized by good solubility in all media, which is indicated by the free energy of solvation value (ΔGsolv, Table S7). For each molecule, the behavior of ΔGsolv as a function of polarity is non-monotonic. The highest solubility is observed in MeCN, the lowest in DMSO for PK1 and PK2 and in toluene for PK3. The arrangement of molecules in terms of this quantity remains analogous to the NLO parameters. The introduction of an aldehyde group into the PK1 system results in a decrease in the free energy of solvation value, and the difference ${\Delta \Delta G}_{solv}^{PK2-PK1}(Tol)$ is 4.02 kcal/mol, while ${\Delta \Delta G}_{solv}^{PK2-PK1}(MeOH)$ is only 0.02 kcal/mol. The average value of ${\Delta \Delta G}_{solv}^{PK3-PK2}$ is 8.03 kcal/mol, with the highest increase in solubility for PK3 relative to PK2 is observed in MeCN (10.37 kcal/mol), and the lowest in toluene (3.90 kcal/mol).

#### 2.4.2. Biological Characteristics

**Figure 4:**
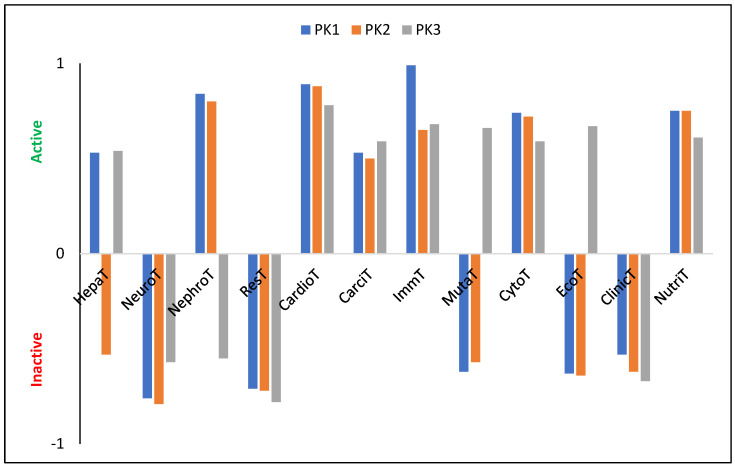
Characterization of theoretical biological properties. HepaT—Hepatotoxicity; NeuroT—Neurotoxicity; NephroT—Nephrotoxicity; ResT—Respiratory toxicity; CardioT—Cardiotoxicity; CarciT—Carcinogenicity; ImmT—Immunotoxicity; MutaT—Mutagenicity; CytoT—Cytotoxicity; EcoT—Ecotoxicity; ClinicT—Clinical toxicity; NutriT—Nutritional toxicity.

The tested molecules are characterized by a relatively high LogP value: 7.19, 8.12, and 8.53 (±0.25) for PK1, PK2, and PK3, respectively. Since these values exceed five, they suggest, in accordance with Lipinski’s [41,42] rule of five, that the penetration of these derivatives through cell membranes may be impeded, potentially reducing their ability to bind to enzymes or receptors at their site of action. A substance is considered to be not bioaccumulative if it has a BCF less than 1, bioaccumulative if it has a BCF from 1 to 5 and very bioaccumulative if it has a BCF greater than 5. The calculated LogBCF values (PK1 = 1.79, PK2 = 1.04, PK3 = −0.08) suggest that only PK3 should be considered as a molecule with no bioaccumulation potential, as it is readily excreted from living organisms via urine. In contrast, the slightly elevated values for PK1 and PK2 imply a possible affinity for lipids, indicating these compounds may preferentially concentrate in lipid-rich tissues rather than in aqueous environments like the cytosol. The PK1 derivative is characterized by relatively intermediate metabolism by CYP450-2D6 (probability (P) is $P^{PK1}=$ 56%) and CYP450-3A4 ($P^{PK1}=$ 55%). The introduction of an aldehyde group into its structure results in the cessation of metabolism via the first cytochrome with a probability of 54%. At the same time, the probability of metabolism by the second cytochrome increases to 77%. The third of the analyzed derivatives does not show any tendency to be metabolized by these cytochromes and in both cases $P^{PK3}$ = 79%. PK1 and PK2 also show a high tendency to be metabolized by CYP450-1A2 ($P^{PK1}=$ 85%, $P^{PK2}=$ 81%) and CYP450-2C19 ($P^{PK1}=$ 74%, $P^{PK2}=$ 75%). In turn, PK3 will not be metabolized by these cytochromes with a probability of 83% and 78%, respectively. Therefore, only PK1 and PK2 derivatives will be satisfactorily removed from tissues and the human body without interacting with other biomolecules and drugs. The theoretically determined oral toxicity LD50 values are 497.80 mg/kg, 1055.00 mg/kg, and 143.20 mg/kg for PK1, PK2, and PK3, respectively. Therefore, these values indicate the need for caution when using PK3, and that the introduction of the –COH group into the system reduces the toxicity of the system by more than two times. For intraperitoneal route of administration, LD50 values are 335.10 mg/kg, 457.10 mg/kg, and 738.50 mg/kg; for intravenous route of administration LD50 values are 60.39 mg/kg, 40.05 mg/kg, and 169.30 mg/kg; for subcutaneous route of administration, LD50 values are 238.70 mg/kg, 981.30, and 1602.00 mg/kg. Moreover, all derivatives show no neurotoxicity, respiratory toxicity, and clinical toxicity (Figure 4). Only PK2 shows no hepatotoxicity. Nephrotoxicity does not show for PK3, and mutagenicity does not show for PK2 and PK3. In turn, all molecules show a relatively high probability of cardiotoxicity, carcinogenicity, immunotoxicity, and cytotoxicity. Moreover, the tested derivatives show many more biological activities (Table S8), proving the possibility of their use as pharmacologically active substances.

The evaluated molecules contain functional groups, specifically –COH (PK2) and –COOH (PK3), which facilitate rapid and efficient conjugation with amino groups, such as those in lysine residues, resulting in the formation of stable and durable bioconjugates with biomacromolecules [43]. To assess the potential of these derivatives as fluorescent markers, molecular docking simulations were conducted using human serum albumin (HSA) and concanavalin A (ConA). HSA is frequently employed as an effective drug delivery system due to its multiple active binding sites, rendering it a critical transporter of various metal ions and organic ligands, including fatty acids, steroids, and a range of pharmaceuticals. For comparative analysis regarding the impact of molecular structure on protein affinity, the PK1 molecule was also included in the simulations, given its potential for interacting with the macromolecule through non-covalent interactions.

**Figure 5:**
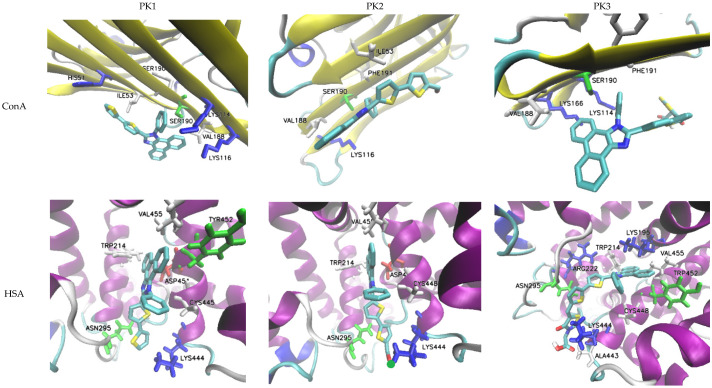
Docking simulation results.

Molecular docking results showed that for all derivatives the zone with the highest affinity for HSA is the active site at CYS448. PK2 has the highest affinity for protein and the affinity value (ΔGb) of −9.3 kcal/mol with an inhibition constant (Ki) of 0.87 mM. At the site of interaction, the fluorescent marker is spatially oriented, positioning itself with the –COH group towards the LYS444 amine group (Figure 5). An H-bond is also formed between the hydrogen atom –NH2 of lysine and the oxygen atom of the aldehyde group. The aromatic cavity is also formed by ASN295, TRP214, ASP451, VAL455, and CYS448 which is cross-linked with the -SH group towards the benzene ring. During spatial alignment, the planar PK2 molecule twists on the bond between imidazole and thiophene (90°), between thiophene rings (30°), and thiophene and –COH (40°). The biocomplex is not stabilized by the formation of cation–π and π–π interactions. PK1 has a slightly lower affinity for HSA with a ΔGb value of −9.1 kcal/mol and Ki = 0.67 mM. In this case, the active center is composed of the same amino acids, and the spatial arrangement of the marker remains analogous to that of PK2. The molecule again directs itself with the oxygen atom of the carboxyl group towards the amino group of LYS444, but without forming an H-bond. Also in this case, spatial alignment causes twisting of the fragment between –COOH and thiophene attached to phenanthroimidazole. Additionally, an interaction between phenanthroimidazole and LYS195 is observed. The system is not stabilized by the formation of cation–π and π–π interactions. ConA is another popular molecular receptor for fluorescence-based glucose detection. This protein contains four binding sites for glucose and competitively binds to glucose in biosensor schemes. Usually, ConA is bound to an existing labeled carbohydrate derivative but is displaced from the molecule when glucose preferentially binds to it. In this case, the PK1 and PK2 derivatives show the same protein affinity value of −5.7 kcal/mol with inhibition constant values of 0.69 mM and 0.74 mM, respectively. The ΔGb value for PK3 drops to −5.3 kcal/mol. In any case, the bioconjugates are not stabilized by the formation of H-bond, cation–π, and π–π interactions. Upon interaction with the biomolecule, PK1 orients itself with the phenanthroimidazole group towards LYS116, while PK2 and PK3 orient with reactive groups towards -NH2 lysine. In the case of PK2, there is competition between LYS116 and LYS114 to form a biocomplex.

### 2.5. Biological Properties

#### 2.5.1. Cytotoxicity

**Figure 6:**
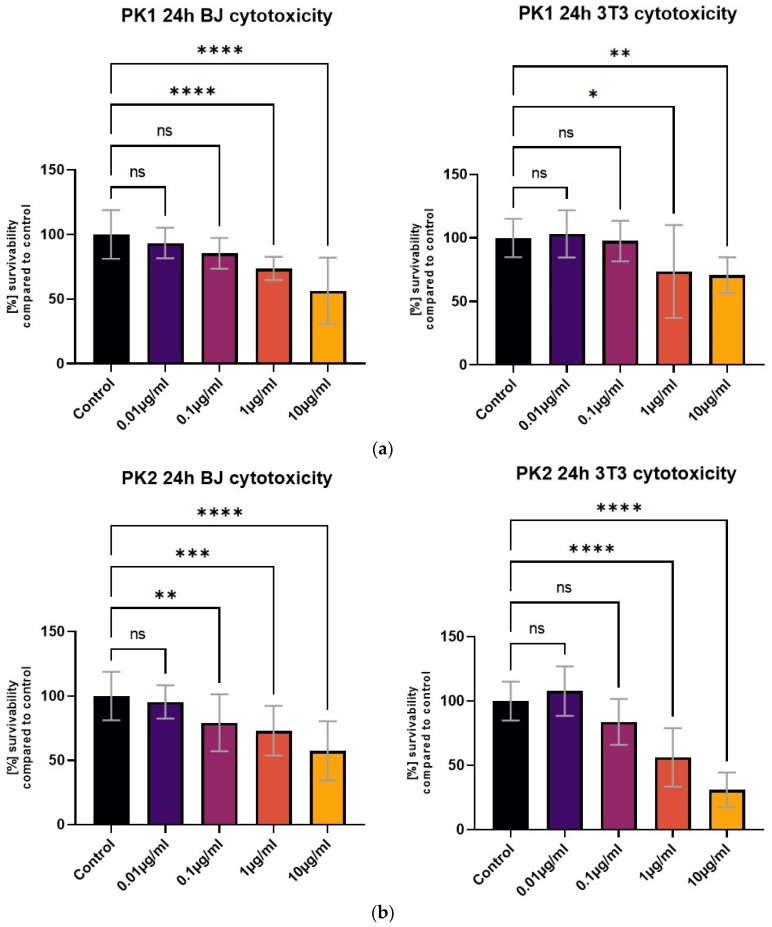

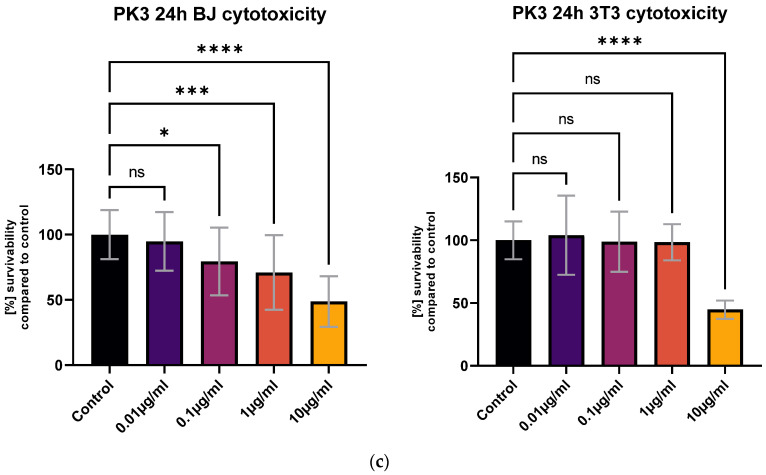
The BJ and 3T3 MTT assay results after 24 h of exposure to various concentrations of (**a**) PK1, (**b**) PK2, and (**c**) PK3. Results presented as [%] of survivability of untreated control. Results, significantly different from that of the control, are marked with [*,**,***,****]. Statistical analysis used—one-way ANOVA with Dunnett’s post hoc test (CI at 95%).

All tested compounds showed some level of cytotoxicity towards the cell lines within the tested concentration range, but none of them were highly cytotoxic, as shown in Figure 6. Differences in cytotoxicity were present between the two tested cell lines; the 3T3 cell line was less sensitive to all of the tested compounds, and was least affected by the PK3 compound. No statistically significant differences between the 1 μg/mL concentration and the control were detected with the MTT assay (Figure 6c). At the same time, the BJ cell line metabolic activity was only statistically unaffected by the lowest 0.01 μg/mL PK3 concentration. The viability of both tested cell lines never dropped below 58% for the 0.1 μg/mL PK3 solution and was never below 68% for 0.1 μg/mL PK1 and PK2.

#### 2.5.2. Imaging of Fixed Stained Cells

**Figure 7:**
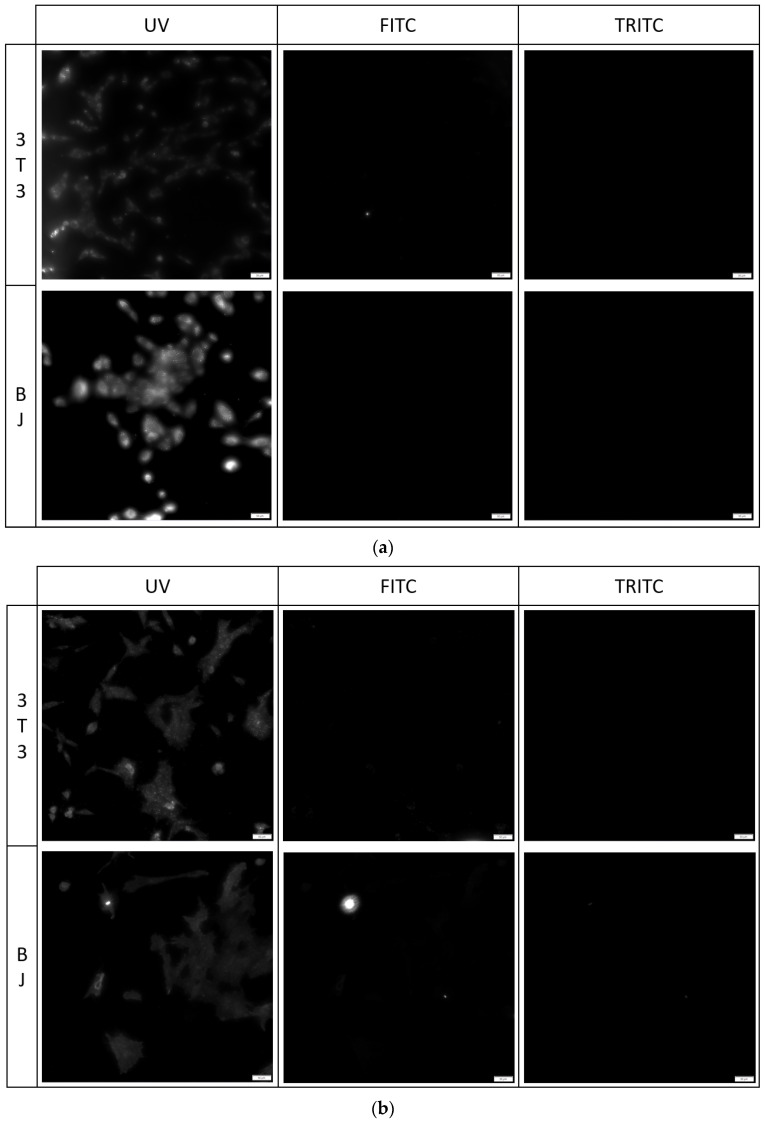
3T3 and BJ cells after 24-hour incubation with 1 μg/mL PK1 fixed with (**a**) 4% formaldehyde or (**b**) 96% ethanol. Cells were simultaneously imaged in three different channels (UV, FITC, TRITC). All cells in the UV channel were stained.

**Figure 8:**
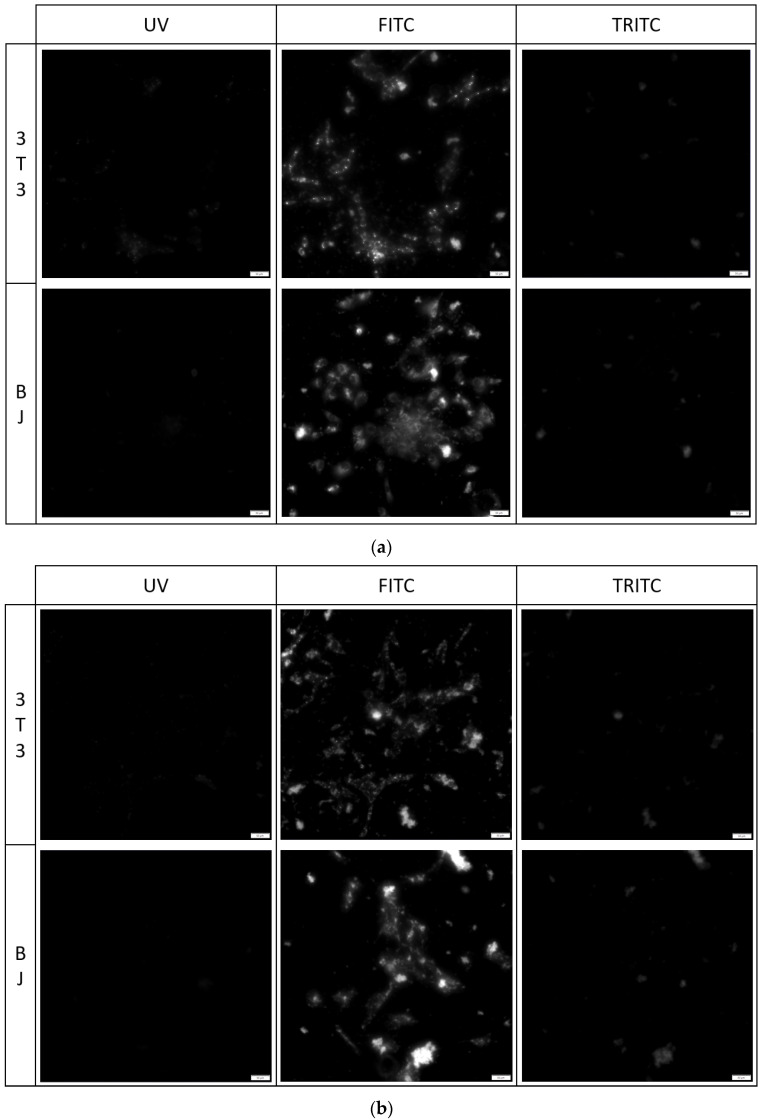
3T3 and BJ cells after 24-hour incubation with 1 μg/mL PK2 fixed with (**a**) 4% formaldehyde or (**b**) 96% ethanol. Cells were simultaneously imaged in three different channels (UV, FITC, TRITC). All cells in the FITC channel were stained.

**Figure 9:**
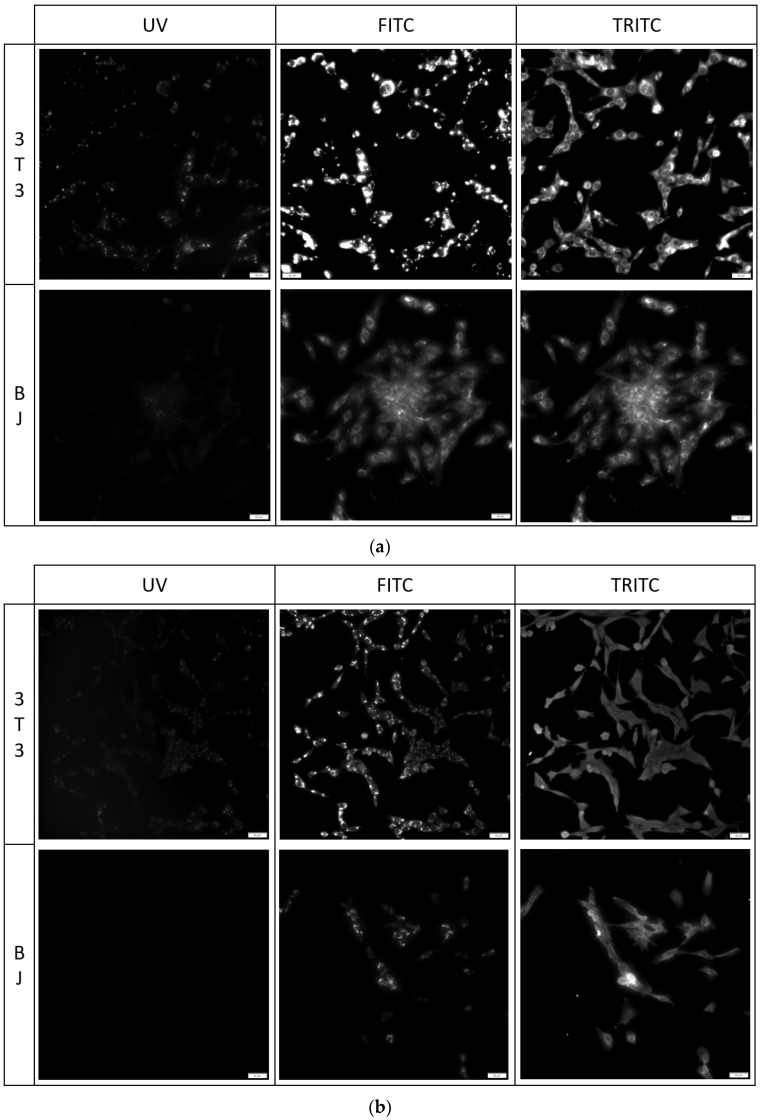
3T3 and BJ cells after 24-hour incubation with 1 μg/mL PK3 fixed with (**a**) 4% formaldehyde or (**b**) 96% ethanol. Cells were simultaneously imaged in three different channels (UV, FITC, TRITC). All cells in the FITC and TRITC channel were stained.

The fixation method had a minor effect on the staining performance of the tested compounds. No difference was found between the staining performance and the tested cell line exposed to the tested compounds (Figure 7, Figure 8 and Figure 9). Fluorescence of PK1 stained cells after fixation was detected only in the UV channel (Figure 7), PK2 only in the FITC channel (Figure 8), and PK3 showed fluorescence in all three tested channels, with the weakest being in the UV channel (Figure 9). Regardless of the compound, images taken from the UV channel shown scattered aggregates of the stain residing on the top side of the cells. On the contrary, PK3 was able to penetrate into the cells as shown in the TRITC channel.

#### 2.5.3. Cell Imaging after Long-Term Exposition

**Figure 10:**
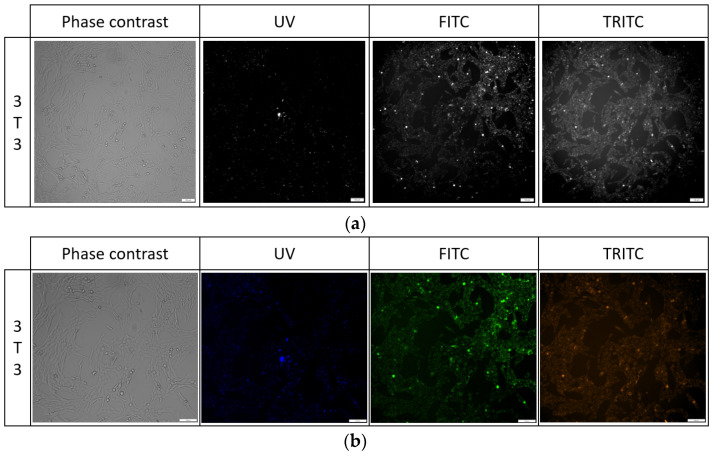
Cell morphology of 3T3 cell line after 24 h exposure to PK3 compound in all tested channels in black and white images (**a**) and color (**b**).

Cells stained for 1 h with 1 μg/mL PK3 exhibited fluorescence in the FITC and TRITC channels, but due to high background noise, it was not possible to take images for analysis. Similarly, after 24 h of exposure, the background noise was at a high level, and a PBS wash was required to reduce it. For PK1 and PK2, cells were unstained after 1 h of incubation, but high background noise was present. No sample fluorescence was detected in the unstained control. For the PK1 compound, cell fluorescence was only visible in the UV channel; for the PK2 in the UV and FITC channels, and for the PK3 compound, the fluorescence of cells was detected in the UV, FITC, and TRITC channels (Table 3). Additionally, images of all samples were taken from the 647 channel, but no fluorescence was detected in this channel. For the 24 h exposure to the tested compounds, we observed no changes in cell morphology for either 3T3 or BJ cell lines while using the concentrations listed in Figure 10 and Table 3.

#### 2.5.4. Antimicrobial Activity

The antimicrobial activity of the newly synthesized compounds PK1, PK2, and PK3 was tested towards 20 reference microorganisms belonging to both bacteria and fungi: Gram-positive bacteria (nine strains), Gram-negative rods (five strains), and yeasts from *Candida* spp. (six strains).

As presented in Table 4 and Table 5, these compounds showed some antimicrobial effect at MICs in the range 500–2000 µg/mL. Among tested bacteria, only four strains of Gram-positive microorganisms, *Staphylococcus aureus* (one of the MSSA strains), *Enterococcus faecalis, Bacillus subtilis* and *Bacillus cereus*, indicated sensitivity to PK1 and PK2 compounds, with high MIC values (1000–2000 µg/mL) and MBC ≥ 2000 µg/mL (MBC/MIC ≥ 1 or > 2). In the case of *B. cereus*, activity of PK2 was mild (MIC = 1000 µg/mL, MBC/MIC = 1). The remaining strains, including also Gram-negative bacteria, were insensitive to both compounds. In turn, substance PK3 showed a slightly stronger antibacterial effect than PK1 and PK2. The activity of this compound towards all reference bacteria, except *Pseudomonas aeruginosa* (MIC = 2000 µg/mL and MBC > 2000 µg/mL), ranged from 500 to 1000 µg/mL (MBC > 2000 µg/mL). PK3 showed moderate antimicrobial effect against one of the MSSA strains and both strains of *Bacillus* spp. (MIC = 500 µg/mL) or moderate bioactivity towards the remaining bacteria (MIC = 1000 µg/mL). MBC/MIC ratios were >2 or >4 in the case of Gram-positive bacteria and >1 or >2 for Gram-negative rods.

The antifungal effect of PK1–PK3 compounds was similar and not high. Among yeasts belonging to reference strains from *Candida* spp., *Candida parapsilosis* was the most sensitive to the tested substances. The compounds, especially PK2 and PK3, showed moderate activity (with MIC = 500 µg/mL, MFC = 2000 µg/mL, and MFC/MIC = 4) and PK1 mild effect (MIC = 1000 µg/mL, MFC = 2000 µg/mL, and MFC/MIC = 2) against these yeasts. The MIC values of compounds ranged from 1000 to 2000 µg/mL toward remaining strains. In turn, the MFCs were the same or 2-fold higher than the MIC, indicating an MFC/MIC index = 1–4 and its fungicidal effect. In the case of *C. krusei*, MFC values were >2000 µg/mL and MFC/MIC index > 2) (Table 5).

## 3. Materials and Methods

The Supplementary Materials contain spectra and analytical results. Moreover, data on materials, methods, and experiments are presented in Supplementary Materials [5,14,27,28,38,44,45,46,47,48,49,50,51,52,53,54,55,56,57,58,59,60,61,62,63]. Abbreviations: AV—average value, Tol—toluene, CHCl_3_—chloroform, MeOH—methanol, MeCN—acetonitrile, DMSO—dimethyl sulfoxide.

## 4. Conclusions

As a result of the research, three phenanthro[9,10-d]-imidazole derivatives (PK1–PK3) were obtained and tested for their properties and applications. PK1 was synthesized by condensation of 2,2′-bithiophene-5-carboxaldehyde, aniline, phenanthrenequinone, and ammonium acetate, with a yield of 74%. Then, the obtained PK1 was subjected to a formylation reaction using N,N-dimethylformamide and phosphorus(V) oxychloride, thanks to which PK2 was synthesized with a yield of 33%. It is worth emphasizing that no direct formylation reaction of phenanthro[9,10-d]-imidazole derivatives has been described in the literature. Finally, PK2 was used for the Knoevenagel condensation reaction with rhodanine-3-acetic acid and ammonium acetate. This made it possible to obtain PK3 with a yield of 78%. Interestingly, measurements of thermal parameters showed that all of the tested derivatives (PK1–PK3) exhibit high thermal stability above 300 °C. The influence of the functional group in this aspect is insignificant. Luminescence studies were successfully performed in five solvents. In both absorption and emission measurements, the influence of the functional group was significant. Modifying the 2,2′-bithiophene substituent causes a significant bathochromic shift consistent with the order PK1 < PK2 < PK3. This can be attributed to the presence of an electron-withdrawing aldehyde group (–CHO) in PK2 and an electron-withdrawing heterocyclic rhodanine-3-acetic acid ring in PK3. Quantum yields were determined for all derivatives. PK1 had the highest quantum yield (26.8%). For PK2 it was 14.5% and for PK3 only 0.6%. The presented results indicate a change in emission from the locally excited singlet emission (^1^LE) state for PK1 to typical ICT emission for PK3. Luminescence studies were enriched with DFT/TDDFT calculation results. Preliminary theoretical studies were performed to assess biological activity. Furthermore, molecular docking simulations were performed using human serum albumin (HSA) and concanavalin A (ConA) to assess these derivatives’ potential as fluorescent markers. All compounds exhibited the staining potential of live cells cultured in an in vitro environment. The staining performance was unaffected by the cell line, and thus, we achieved proper staining of both mice and human cell lines. PK3 has proven to be the most attractive of the tested compounds, thanks to its staining potential for live cells and retention after fixation. We could also see above 70% viability after 24 h exposure to 1μg/ml PK3 and achieve usable staining performance for cell imaging. Staining with PK2 also yielded high cell viability at the concentration which gave adequate staining performance. All compounds were compatible with formaldehyde and ethanol fixation methods, as we could image the stained cells after fixation without issues at the same concentrations of the tested compounds as when no fixation method was used. Our results showed some antibacterial and antifungal effect of the newly synthesized compounds PK1–PK3. Among them, PK3 showed the highest antimicrobial effect, especially against Gram-positive bacteria. Its activity was moderate or mild towards these microorganisms.

## Figures and Tables

**Table 1:** Thermal data of the PK series.

Code	T_m_^a^ [°C]	T_5_^b^ [°C]	T_10_^b^ [°C]	T_max_^c^ [°C]
PK1	265	313	341	393
PK2	267	332	348	378
PK3	355	331	347	362

^a^ T_m_ is the melting point. ^b^ T_5_ and T_10_ are temperature at 5% and 10% weight loss, respectively. ^c^ Temperature of maximum decomposition rate.

**Table 2:** Collected data on photophysical properties of the PK series.

Compound	Solvent	λ_abs_ [nm]	λ_em_ [nm]	Stock Shift * [nm]	QY [%]	Chromaticity Coordinates u’, v’
PK1	Toluene	384	437; 461	53	-	0.12; 0.29
	CHCl_3_	382	441; 464	59	-	0.12; 0.31
	MeCN	379	440sh; 458	61	-	0.12; 0.29
	DMSO	386	445; 465	59	26.8	0.11; 0.32
	MeOH	372	444; 465	72	-	0.13; 0.28
PK2	Toluene	421	495; 513sh	74	-	0.08; 0.54
	CHCl_3_	420	531	111	-	0.13; 0.56
	MeCN	411	547	136	-	0.16; 0.56
	DMSO	418	553	135	14.5	0.18; 0.56
	MeOH	406	564	158	-	0.22; 0.56
PK3	Toluene	379; 492	581	89	-	0.30; 0.55
	CHCl_3_	380; 497	608	111	-	0.33; 0.54
	MeCN	374; 482	571sh; 607	125	-	0.29; 0.55
	DMSO	379; 493	614	121	0.6	0.34; 0.54
	MeOH	371; 471	585	114	-	0.29; 0.55

* λem − λabs. toluene (ε_Tol_ = 2.38), chloroform (ε_CHCl3_ = 4.81), methanol (ε_MeOH_ = 33.0), acetonitrile (ε_MeCN_ = 36.6), dimethyl sulfoxide (ε_DMSO_ = 47.24).

**Table 3:** Table presenting the minimal concentrations of tested compounds resulting in visible fluorescence of 3T3 and BJ cell lines after 24-hour exposure. The table also provides channels in which the fluorescence was detected.

	PK1	PK2	PK3
3T3	1 μg/mL UV	0.1 μg/mL UV/FITC	1 μg/mL UV/FITC/TRITC
BJ	1 μg/mL UV	0.1 μg/mL UV/FITC	1 μg/mL UV/FITC/TRITC

**Table 4:** The activity data of the studied compounds PK1–PK3 expressed as MIC, MBC [µg/mL] values, and MBC/MIC ratios against the reference strains of bacteria. The standard antibiotics ciprofloxacin (CIP) and vancomycin (VA *) were used as positive controls.

Species		PK1			PK2			PK3			CIP/VA *		
		MIC	MBC	MBC/MIC	MIC	MBC	MBC/MIC	MIC	MBC	MBC/MIC	MIC	MBC	MBC/MIC
**Gram-positive bacteria**	*Staphylococcus aureus* MRSA ATCC 43300	-	-	-	-	-	-	1000	>2000	>2	0.24	0.24	1
	*Staphylococcus aureus* MSSA ATCC 25923	-	-	-	-	-	-	1000	>2000	>2	0.48	0.48	1
	*Staphylococcus aureus* MSSA ATCC 6538	2000	>2000	>1	2000	>2000	>1	1000	>2000	>2	0.48	0.48	1
	*Staphylococcus aureus* MSSA ATCC 29213	-	-	-	-	-	-	500	>2000	>4	0.48	0.48	1
	*Staphylococcus epidermidis* ATCC 12228	-	-	-	-	-	-	1000	>2000	>2	0.12	0.12	1
	*Enterococcus faecalis* ATCC 29212	2000	>2000	>1	2000	>2000	>1	1000	>2000	>2	0.98 *	1.95 *	2 *
	*Micrococcus luteus* ATCC 10240	-	-	-	-	-	-	1000	>2000	>2	0.98	1.98	2
	*Bacillus subtilis* ATCC 6633	2000	2000	1	2000	2000	1	500	>2000	>4	0.03	0.03	1
	*Bacillus cereus* ATCC 10876	2000	>2000	>1	1000	>2000	>2	500	>2000	>4	0.06	0.12	2
Gram-negative bacteria	*Escherichia coli* ATCC 25922	-	-	-	-	-	-	1000	>2000	>2	0.004	0.004	1
	*Klebsiella* *pneumoniae* ATCC 13883	-	-	-	-	-	-	1000	>2000	>2	0.12	0.12	1
	*Proteus mirabilis* ATCC 12453	-	-	-	-	-	-	1000	>2000	>2	0.03	0.03	1
	*Salmonella* Typhimurium ATCC 14028	-	-	-	-	-	-	1000	>2000	>2	0.06	0.06	1
	*Pseudomonas aeruginosa* ATCC 27853	-	-	-	-	-	-	2000	>2000	>1	0.48	0.98	2

Activity: no bioactivity—MIC > 1000 µg/mL; mild bioactivity—MIC = 501–1000 µg/mL; moderate bioactivity MIC = 126–500 µg/mL. * indicates the use of vancomycin (VA *) as a standard antibiotic.

**Table 5:** The activity data of the studied compounds PK1–PK3 expressed as MIC, MFC [µg/mL] values, and MFC/MIC ratios against the reference strains of fungi. The standard antibiotic nystatin (NY) was used as positive control.

Species		PK1			PK2			PK3			NY		
		MIC	MFC	MFC/MIC	MIC	MFC	MFC/MIC	MIC	MFC	MFC/MIC	MIC	MFC	MFC/MIC
Fungi	*Candida* *albicans* ATCC 10231	2000	2000	1	2000	2000	1	2000	2000	1	0.48	0.48	1
	*Candida* *albicans* ATCC 2091	2000	2000	1	1000	2000	2	1000	2000	2	0.24	0.24	1
	*Candida* *parapsilosis* ATCC 2201	1000	2000	2	500	2000	4	500	2000	4	0.24	0.48	2
	*Candida* *glabrata* ATCC 90030	2000	2000	1	2000	2000	1	2000	2000	1	0.24	0.48	2
	*Candida* *krusei* ATCC 14243	1000	>2000	>2	2000	>2000	>1	1000	>2000	>2	0.24	0.24	1
	*Candida* *auris* CDC B11903	2000	2000	1	2000	2000	1	2000	2000	1	0.48	0.48	1

Activity: no bioactivity—MIC > 1000 µg/mL; mild bioactivity—MIC = 501–1000 µg/mL; moderate bioactivity MIC = 126–500 µg/mL.

## Data Availability

The original contributions presented in the study are included in the Supplementary Materials; further inquiries can be directed to the corresponding author.

## Supplementary Materials

The following supporting information can be downloaded at: https://www.mdpi.com/article/10.3390/molecules29194703/s1, Figure S1: ^1^H NMR of PK-1; Figure S2: ^13^C NMR of PK-1; Figure S3: ^1^H NMR of PK-2; Figure S4: ^13^C NMR of PK-2; Figure S5: ^1^H NMR of PK-3; Figure S6: ^13^C NMR of PK-3; Figure S7: DSC thermograms of PK1; Figure S8: DSC thermograms of PK2; Figure S9: DSC thermograms of PK3; Figure S10: TGA thermogram—thermal properties of PK series; Figure S11: Comparison of absorption and emission behavior of PK series; Figure S12: UV–Vis spectra recorded in DMSO for 24 h at room temperature; Figure S13: Chromaticity plots of the PK series in various solvents; Figure S14: The Lippert–Mataga plots; Figure S15: HOMO/LUMO plots; Figure S16: The MEP surfaces; Figure S17: Density difference plots; Table S1: The frontier orbital energies in tested solvents for Z-isomers. Values are given in [eV]; Table S2: Linear optical properties (nm); Table S3: Calculated values of dipole moments (in D) for the ground and CT excited state; Table S4: CT parameters for the bright low-lying excited state. Values qCT are given in [e] and DCT in [Å]; Table S5: Nonlinear properties. Values are given in (a.u.); Table S6: Two-photon absorption cross-section. Values $\langle \delta^{OF}\rangle$ are given in [a.u.] and $\sigma_{OF}^{\left(2\right)}$ in [GM]; Table S7: Free energies of solvation (kcal/mol); Table S8: Biological activities (P—Probability; Pred—Prediction ; A—active; InA—inactive).
