# Supplementary material for: The Influence of the Functional Group on the Physicochemical and Biological Properties of New Phenanthro[9,10-d]-Imidazole Derivatives
Source: Molecules. 2024 Oct 4;29(19):4703. doi: 10.3390/molecules29194703 (PMC11477550; doi:10.3390/molecules29194703)
Supplement: Supplementary file 1 [file molecules-29-04703-s001.zip › molecules-3211251-supplementary.pdf]

Supporting Information

The influence of the functional group on the physicochemical and biological properties of new phenanthro[9,10-d]-imidazole derivatives

Slawomir Kula [a]*, Łukasz Kaźmierski [b], Paweł Kalarus [a], Anna Biernasiuk [c], Przemysław Krawczyk [d]

[a] Institute of Chemistry, Faculty of Science and Technology, University of Silesia, Szkolna 9 St., 40-007 Katowice, Poland; slawomir.kula@us.edu.pl, pawelkalarus2000@gmail.com

[b] Nicolaus Copernicus University, Collegium Medicum, Faculty of Medicine, Department of Oncology, Radiotherapy and Oncological, M. Curie Skłodowskiej 9, 85-094 Bydgoszcz, Poland; lukasz.kazmierski@cm.umk.pl

[c] Department of Pharmaceutical Microbiology, Faculty of Pharmacy, Medical University of Lublin, 20-093 Lublin, Poland; anna.biernasiuk@umlub.pl

[d] Nicolaus Copernicus University, Collegium Medicum, Faculty of Pharmacy, Department of Physical Chemistry, Kurpińskiego 5, 85-950 Bydgoszcz, Poland; przemekk@cm.umk.pl

Table of Contents

1. **Materials** 2
2. **Measurements and general methods** 2
3. **Synthesis and NMR spectra** 5
4. **Thermal properties** 11
5. **Optical properties** 12
6. **DFT calculations - methodology** ....Error! Bookmark not defined.5
7. **Literature** 22

1. Materials

9,10-Phenanthrenedione (≥99%, Merck-Sigma-Aldrich), Ammonium acetate (puriss. p.a., ACS reagent, reag. Ph. Eur., ≥98%, Merck-Sigma-Aldrich), 2,2'-Bithiophene-5-carboxaldehyde (98%, TCI), Aniline (99%, Merck-Sigma-Aldrich), Rhodanine-3-acetic Acid (98%, TCI), Phosphorus(V) oxychloride (Merck-Sigma-Aldrich), Glacial acetic acid (99,5% pure p. a, Chempur), N,N-Dimethylformamide (pure, Karpinex), Chloroform-d ($CDCl_3$, 99.8 atom % D, Merck-Sigma-Aldrich), Dimethyl sulfoxide-d_6 (DMSO-d_6, 99.9 atom % D, Merck-Sigma-Aldrich), Acetonitrile (for HPLC-GC, ≥99.8% (GC), Merck-Sigma-Aldrich), Chloroform (for HPLC, ≥99.8%, amylene stabilized, Merck-Sigma-Aldrich), Methanol (for HPLC, ≥99.9%, Merck-Sigma-Aldrich), DMSO (pure for analysis, Chempur), Toluene (pure for analysis, Chempur).

2. Measurements and general methods

Bruker Avance 400 instrument and Bruker Avance 500 instrument were used to record the NMR spectra in $CDCl_3$ and DMSO-d_6 (as solvents). Differential scanning calorimetry (DSC) and thermogravimetric analysis (TGA) were performed on the following devices: TA – DSC 25 and TA – TGA 55. UV/Vis spectra were recorded with a Biosens model UV 5600 UV/Vis spectrophotometer. Photoluminescence emission spectra were acquired using Hitachi Fluorescence Spectrophotometer F-7100.

In vitro antimicrobial assay

The examined compounds **PK1, PK2** and **PK3** were screened *in vitro* for antibacterial and antifungal activities using the broth microdilution method according to European Committee on Antimicrobial Susceptibility Testing (EUCAST) [1] and Clinical and Laboratory Standards Institute guidelines [2] against a panel of reference strains of microorganisms, including Gram-positive bacteria (*Staphylococcus aureus* ATCC 43300 (Methicillin Resistant *S. aureus* – MRSA), *Staphylococcus aureus* ATCC 29213 (Methicillin Susceptible *S. aureus* – MSSA), *Staphylococcus aureus* ATCC 25923 (Methicillin Susceptible *S. aureus* – MSSA, *Staphylococcus aureus* ATCC 6538 (Methicillin Susceptible *S. aureus* – MSSA), *Staphylococcus epidermidis* ATCC 12228, *Enterococcus faecalis* ATCC 29212, *Micrococcus luteus* ATCC 10240, *Bacillus subtilis* ATCC 6633 and *Bacillus cereus* ATCC 10876), Gram-negative bacteria (*Escherichia coli* ATCC 25922, *Klebsiella pneumoniae* ATCC 13883, *Proteus mirabilis* ATCC 12453, *Salmonella* Typhimurium ATCC 14028 and *Pseudomonas aeruginosa* ATCC 9027) and fungi belonging to yeasts (*Candida albicans* ATCC 2091, *Candida albicans* ATCC 10231, *Candida parapsilosis* ATCC 22019, *Candida glabrata* ATCC

90030 and *Candida krusei* ATCC 14243, and *Candida auris* CDC B11903). The microorganisms came from American Type Culture Collection (ATCC) routinely used for the evaluation of antimicrobials, or from Centers for Disease Control and Prevention (CDC). All the used microbial cultures were first subcultured on nutrient agar or Sabouraud agar at 35°C for 18-24 h or 30°C for 24-48 h for bacteria and fungi, respectively.

Samples containing examined compounds **PK1, PK2** and **PK3** were first dissolved in dimethyl sulfoxide – DMSO (20 mg/mL). Subsequently MIC (Minimal Inhibitory Concentration) of these substances was examined by the microdilution broth method, using their two-fold dilutions in Mueller-Hinton broth (for bacteria) and RPMI 1640 broth with MOPS (for fungi) prepared in 96-well polystyrene plates. Final concentrations of the compounds ranged from 2000 to 15.62 µg/mL. Microbial suspensions were prepared in 0.85% NaCl with an optical density of 0.5 McFarland standard. Next bacterial or fungal suspensions were added per each well containing broth and various concentrations of the examined compounds. After incubation (under the same conditions as before), the MIC was assessed spectrophotometric as the lowest concentration of the samples showing complete bacterial or fungal growth inhibition. Appropriate, growth and sterile controls were carried out. The medium with no tested substances was used also as control. The inhibition of microbial growth was judged by comparison with a control culture prepared without any sample tested. Ciprofloxacin, vancomycin or nystatin (Sigma-Aldrich Chemicals, St. Louis, MO, USA) were used as a reference antibacterial or antifungal compounds, respectively.

The MBC (Minimal Bactericidal Concentration) or MFC (Minimal Fungicidal Concentration) are defined as the lowest concentration of the compounds that is required to kill a particular bacterial or fungal species. MBC or MFC was determined by removing the culture using for MIC determinations from each well and spotting onto appropriate agar medium. Next, the plates were incubated. The lowest compounds concentrations with no visible growth observed were assessed as a bactericidal or fungicidal concentrations. All the experiments were repeated three times and representative data are presented [3, 4, 5].

In this study, no bioactivity was defined as a MIC > 1000 µg/ml, mild bioactivity as a MIC in the range 501 – 1000 µg/ml, moderate bioactivity with MIC from 126 to 500 µg/ml, good bioactivity as a MIC in the range 26 – 125 µg/ml, strong bioactivity with MIC between 10 and 25 µg/ml and very strong bioactivity as a MIC < 10 µg/ml [6]. The MBC/MIC or MFC/MIC ratios were calculated in order to determine bactericidal/fungicidal (MBC/MIC ≤ 4,

MFC/MIC ≤ 4) or bacteriostatic/fungistatic (MBC/MIC > 4, MFC/MIC > 4) effect of the tested compounds.

In vitro cell culture

Mouse fibroblasts (3T3/NIH, CRL-1658, ATCC) and human fibroblasts (BJ-5ta, CRL-4001, ATCC) were utilized as model cell lines for testing compound cytotoxicity and staining. Prior to the experiment, the cell lines were maintained in culture for at least five passages. Standard culture conditions were followed: 37°C, 5% CO2, and 98% humidity. The 3T3 cells were cultured in DMEM/F12K medium (Corning USA), while Medium 199 (Corning USA) was used for BJ-5ta cells. All media were supplemented with 10% FBS (Corning USA) and the recommended concentration of antibiotics.

Stock reagent preparation

The stock solution of the staining reagents was prepared in cell culture certified DMSO (Dimethyl sulfoxide, BioUltra purity, Merck, USA). After dissolving the compounds, they were centrifuged for 5 minutes at 1000xg and filtered using a 0,22 µm syringe filter (PES Millex-GP).

Cytotoxicity assay and Live-cell staining

To assess the cytotoxicity of the tested compounds (PK1, PK2, and PK3), we conducted a 24-hour exposure, starting with a concentration of 10 µg of the substance per 1 ml of culture medium and diluting down to 0.01 µg per 1 ml of the tested compounds. Following the exposure, an MTT assay was performed. Prior to exposure, cells were seeded in 96-well microplates (Nunc FB TC) at a density of 6,000 cells per well and pre-incubated for 24 hours. After pre-incubation, the medium was removed, and the wells were washed twice with PBS. The compounds were pre-diluted in culture medium across a concentration range (10 µg/ml to 0.01 µg/ml). After the exposure period, the compounds were discarded, and the wells were washed twice with PBS. The MTT assay was then immediately carried out.

To assess the live-cell staining capabilities of the tested compounds, observations were made during the cell exposure phase. The progress of cell staining was monitored using a fluorescence microscope (IX83, Olympus) at 1 hour and 24 hours after staining.

The MTT assay procedure begins with the addition of MTT reagent (Thiazolyl Blue Tetrazolium Bromide, Sigma) at a concentration of 1 mg/ml, diluted in DMEM medium without phenol red. After 2 hours of incubation, the MTT solution is removed, and 100 µl of DMSO is

added to each well. Absorbance is then measured at 570 nm using a multi-plate reader (Multiskan Sky, Thermo, USA). The results are presented as a percentage of cell viability based on absorbance readings compared to the untreated control.

Imaging of fixed, stained cells

We evaluated the impact of different fixation methods on staining effectiveness by fixing cells with 4% methanol-free formaldehyde and with ice-cold 96% ethanol for 15 minutes. Following two PBS washes, the cells were mounted onto glass slides using a hardening medium (Vectashield Vibrance HS). Fluorescence imaging of all samples was conducted using a fully motorized, inverted IX83 microscope (Olympus, Japan) equipped with an Orca Flash 4.0 LT3 monochromatic camera (Hamamatsu, Japan). Images captured in this hardware configuration were monochromatic, if any color images are presented those are a result of pseudo-color post processing. To minimize photobleaching and provide narrow excitation wavelengths, an LED light source (pE-300 ultra, Colled, Great Britain) was used. Custom-made filter cubes are equivalent to U-FUNA, U-FBNA, U-FGW, and U-FMCHE (Olympus, Japan) were utilized for imaging in the UV, FITC, TRITC, and 647/Cy3 channels. The final achieved excitations and emissions for this hardware configuration were: UV (ex: 350-360nm, em: 416-479nm), FITC (ex: 460-495nm, em: 510-550nm), TRITC (ex: 550-585, em: 600-700nm). Focusing was achieved with a semi-automated system using laser autofocus (Z-Drift Compensation Unit, Olympus, Japan).

3. Synthesis and NMR spectra

Synthetic procedures

2-(2,2’-bithiophen-5-yl)-1-phenyl-1*H*-phenanthro[9,10-*d*]-imidazole (PK1)

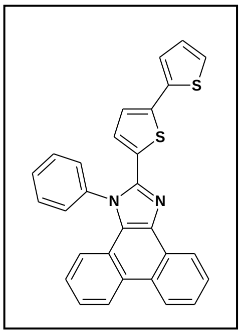

Synthetic procedure based on previously conducted research [7]. A mixture of 2,2’-bithiophene-5-carboxaldehyde (6.00 mmol), aniline (2,79 g, 30.00 mmol), phenanthrenequinone (1,25 g, 6.00 mmol), ammonium acetate (1,85 g, 24.0 mmol) and acetic acid (45 mL) was heated at reflux (135°C) under argon atmosphere. After 24h, the solid was collected by filtration and washed with an acetic acid/water mixture (1:1, 100 mL) and water. The crude product was purified by column chromatography using silica gel and an eluent as a mixture of hexane: dichloromethane (1:3). **PK1** was obtained as yellow solid with 74% yield. **^{1}H NMR (400 MHz, $CDCl_3$) δ** 8.85 (d, J = 7.9 Hz, 1H), 8.75 (d, J = 8.3 Hz,

1H), 8.69 (d, J = 8.3 Hz, 1H), 7.77 – 7.62 (m, 7H), 7.52 – 7.47 (m, 1H), 7.25 – 7.13 (m, 4H), 7.02 (dd, J = 5.1, 3.6 Hz, 1H), 6.94 (d, J = 3.9 Hz, 1H), 6.60 (d, J = 3.9 Hz, 1H). **^{13}C NMR (100 MHz, $CDCl_3$) δ** 145.70, 139.26, 138.57, 137.83, 137.00, 131.92, 130.81, 130.76, 129.41, 128.48, 128.46, 128.10, 127.59, 127.40, 127.09, 126.51, 125.88, 125.11, 125.07, 124.38, 124.28, 124.13, 123.22, 123.08, 122.95, 120.73.

5’-(1-phenyl-1H-phenanthro[9,10-d]imidazol-2-yl)-2,2’-bithiophene-5-carbaldehyde (PK2)

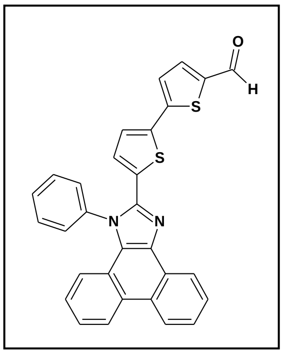

0,92 g (2 mmol) 2-(2,2’-bithiophen-5-yl)-1-phenyl-1*H*-phenanthro[9,10-*d*]-imidazole (**PK1**) and 20 ml N,N-dimethylformamide were added to a 50 ml round-bottom flask.The resulting reaction mixture was cooled in an ice bath to 0°C, and then 1.20 ml (12 mmol) of phosphorus(V) oxychloride was added dropwise. After 1 hour, the cooling bath was removed and the reaction mixture was slowly heated to 90°C. Heating at the set temperature and intensive stirring were continued for another 48 hours. After this time, the reaction mixture was cooled to room temperature. The crude product was precipitated by adding water, filtered and then washed thoroughly with water. In the next step, column chromatography was performed using silica gel (SiO_2) as the solid phase and dichloromethane as the eluent. Finally, **PK2** was obtained as an orange solid with a yield of 33%. **^{1}H NMR (500 MHz, DMSO) δ** 9.90 (s, 1H), 8.92 (d, J = 8.3 Hz, 1H), 8.87 (d, J = 8.4 Hz, 1H), 8.65 (dd, J = 7.9, 1.1 Hz, 1H), 8.01 (d, J = 4.0 Hz, 1H), 7.89 – 7.83 (m, 5H), 7.81 – 7.78 (m, 1H), 7.73 – 7.68 (m, 1H), 7.59 – 7.55 (m, 2H), 7.44 (d, J = 4.0 Hz, 1H), 7.37 – 7.33 (m, 1H), 7.09 (d, J = 8.3 Hz, 1H), 6.51 (d, J = 4.0 Hz, 1H). **^{13}C NMR (125 MHz, DMSO) δ** 183.96, 144.47, 144.39, 141.79, 139.16, 137.55, 136.73, 136.45, 134.05, 131.22, 131.04, 129.03, 128.68, 128.17, 127.86, 127.62, 127.44, 127.36, 126.84, 126.21, 126.08, 125.93, 125.61, 124.59, 123.76, 122.11, 122.05, 120.10.

2-[5-((5’-(1-phenyl-1H-phenanthro[9,10-d]imidazol-2-yl)-2,2’-bithiophene-5-yl)methylene)-4-oxo-2-thioxo-thiazolidin-3-yl]acetic acid (PK3)

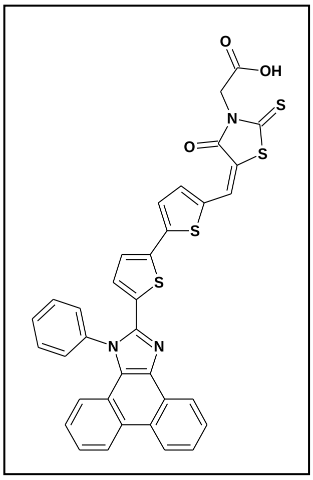

0,32 g (0,70 mmol) 5'-(1-phenyl-1H-phenanthro[9,10-d]imidazol-2-yl)-2,2'-bithiophene-5-carbaldehyde (**PK2**), 0,16 g (0,80 mmol) rhodanine-3-acetic acid, 0,14 g (1,80 mmol) ammonium acetate and 10 ml glacial acetic acid were added to a 50 ml round-bottom flask. The resulting reaction mixture was heated at 135°C for 24 hours with constant stirring. After this time, the reaction mixture was cooled to room temperature, and the crude product was precipitated with the addition of water. Then, it was filtered and washed thoroughly with water. This procedure produced **PK3** as a brown solid with a yield of 78%. **^{1}H NMR (500 MHz, DMSO) δ** 8.90 (d, *J* = 8.4 Hz, 1H), 8.86 (d, *J* = 8.5 Hz, 1H), 8.64 (dd, *J* = 7.9, 1.3 Hz, 1H), 8.09 (s, 1H), 7.90 – 7.83 (m, 5H), 7.81 – 7.77 (m, 1H), 7.75 (d, *J* = 4.0 Hz, 1H), 7.72 – 7.68 (m, 1H), 7.58 – 7.53 (m, 2H), 7.38 (d, *J* = 4.0 Hz, 1H), 7.36 – 7.31 (m, 1H), 7.11 – 7.05 (m, 1H), 6.41 (d, *J* = 4.1 Hz, 1H), 4.68 (s, 2H). **^{13}C NMR (125 MHz, DMSO) δ** 191.58, 167.27, 165.85, 144.39, 144.01, 137.82, 137.61, 136.77, 136.50, 136.33, 133.75, 131.19, 131.05, 129.04, 129.00, 128.67, 128.65, 128.16, 127.87, 127.56, 127.39, 127.14, 126.82, 126.79, 126.52, 126.38, 126.19, 126.05, 125.54, 124.54, 123.72, 122.09, 120.08, 118.80, 45.08.

NMR spectra

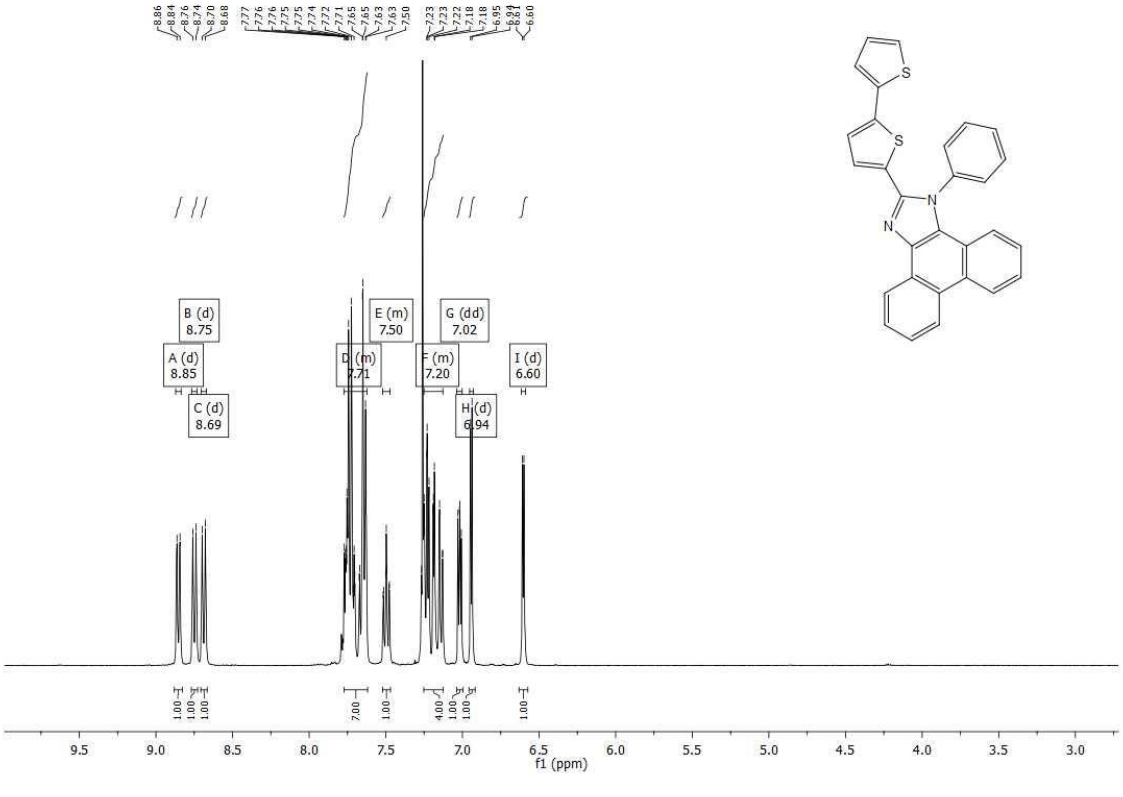

Fig. S1. ^{1}*H NMR of* *PK-1*

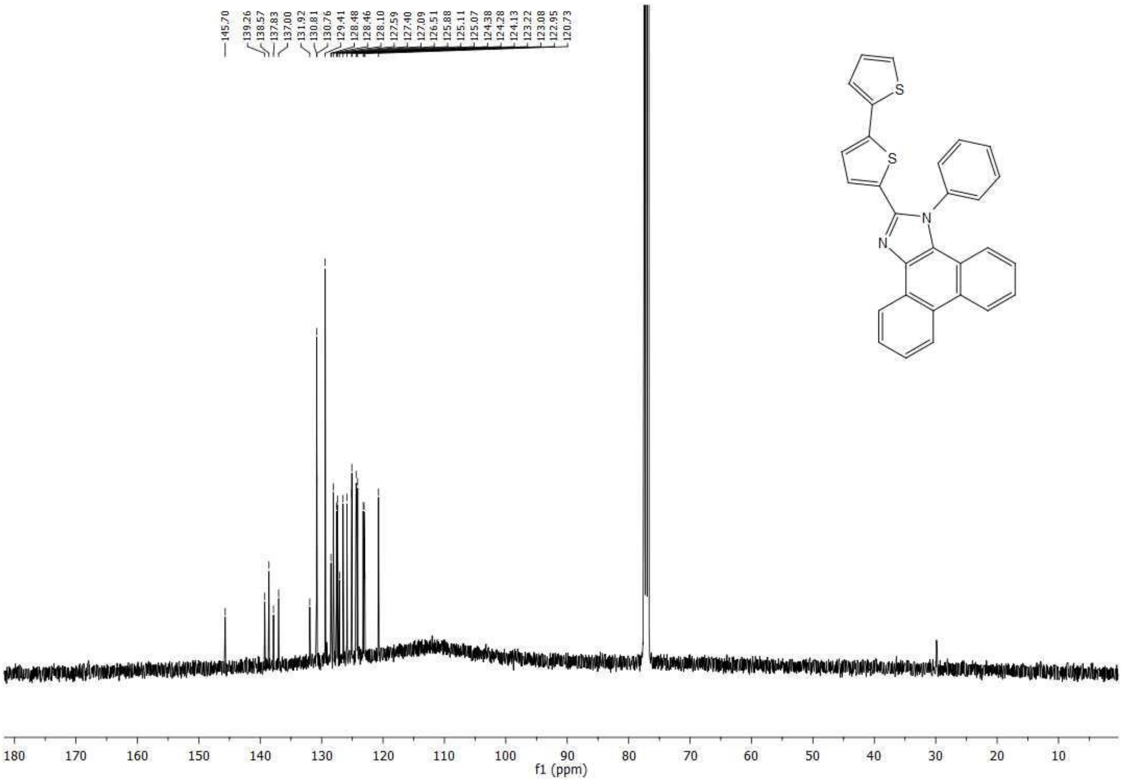

Fig. S2. ^{13}*C NMR of* *PK-1*

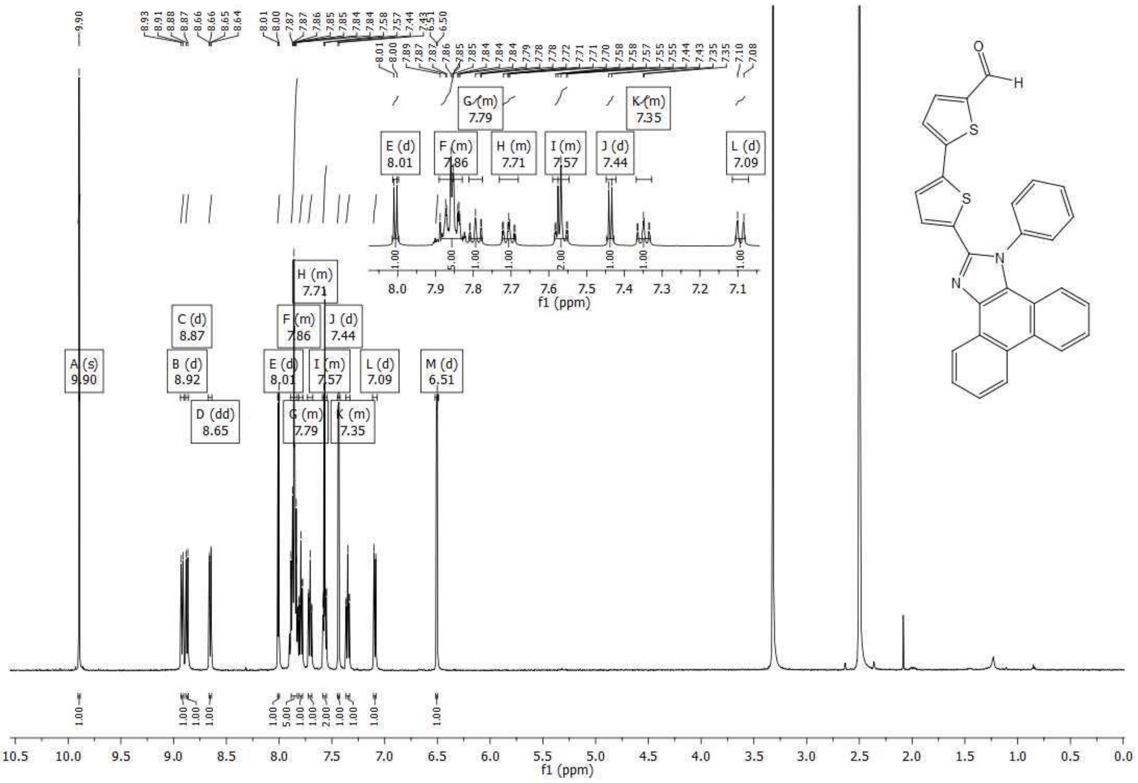

Fig. S3. ^{1}H *NMR of PK-2*

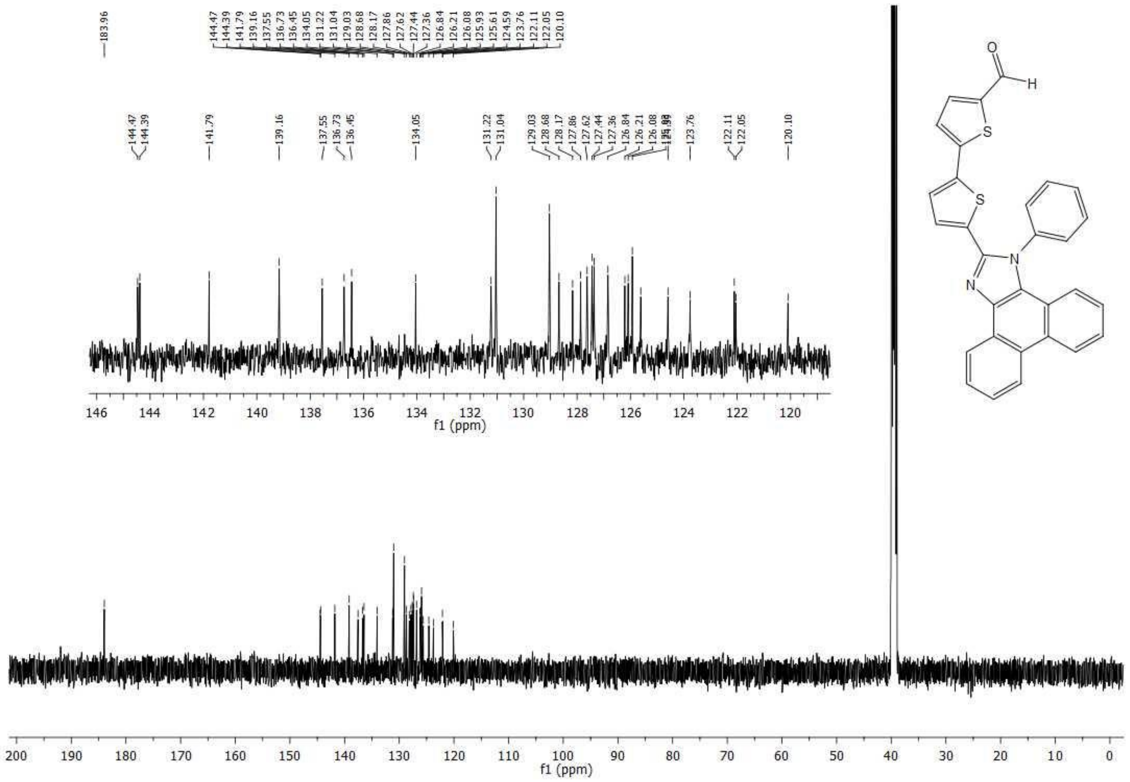

Fig. S4. ^{13}C *NMR of PK-2*

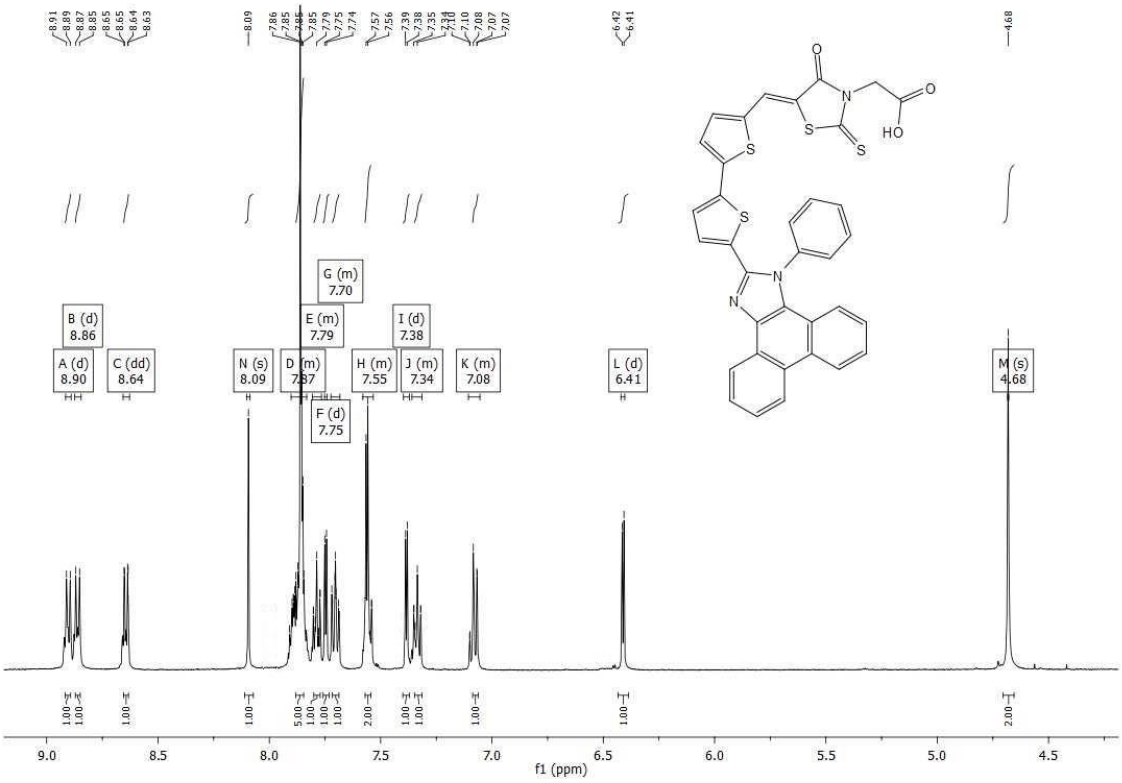

Fig. S5. ***^{1}H NMR of PK-3***

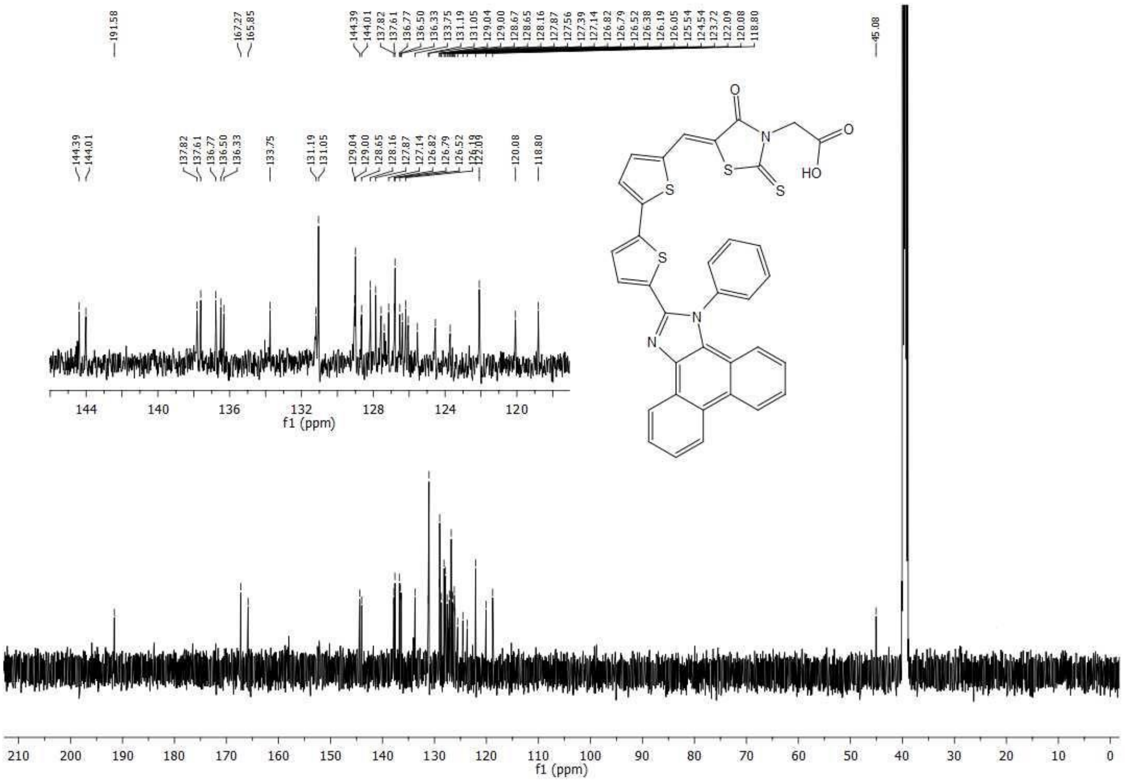

Fig. S6. ***^{13}C NMR of PK-3***

4. Thermal properties

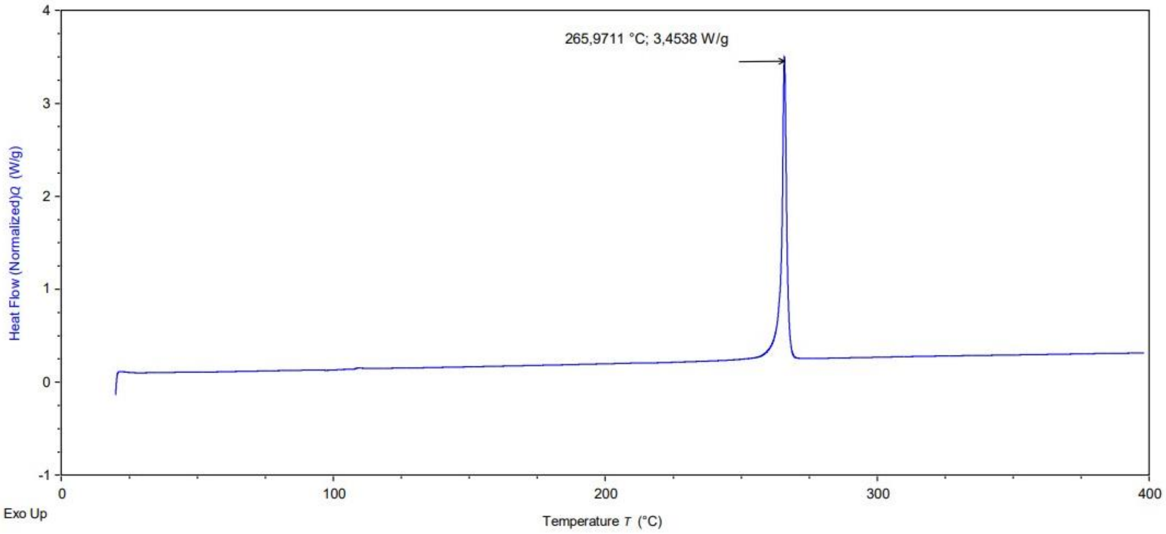

Fig. S7. DSC thermograms of **PK1**

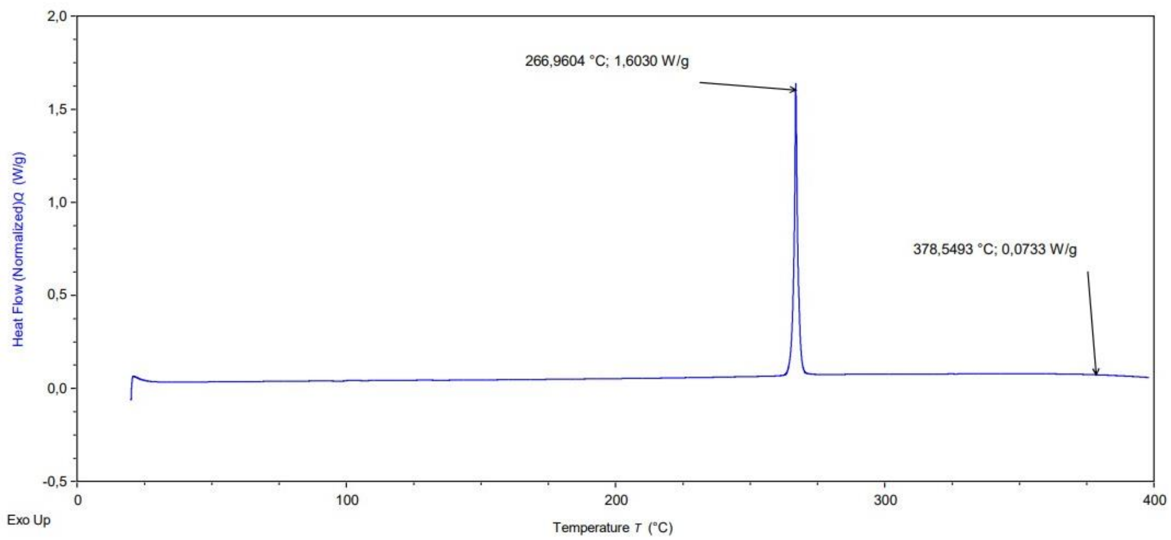

Fig. S8. DSC thermograms of **PK2**

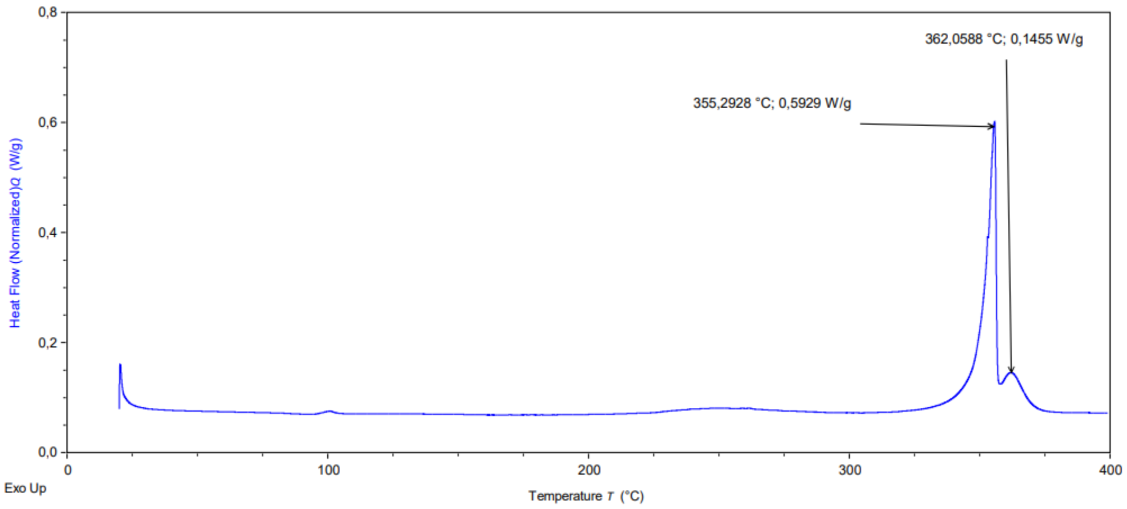

Fig. S9. DSC thermograms of **PK3**

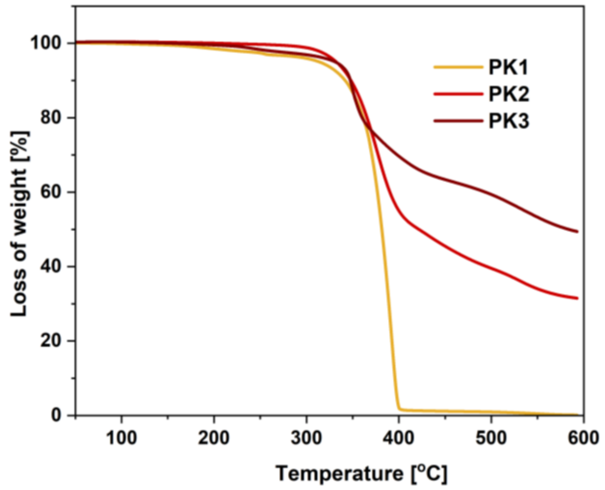

Fig. S10. TGA thermogram - thermal properties of **PK** series

5. Optical properties

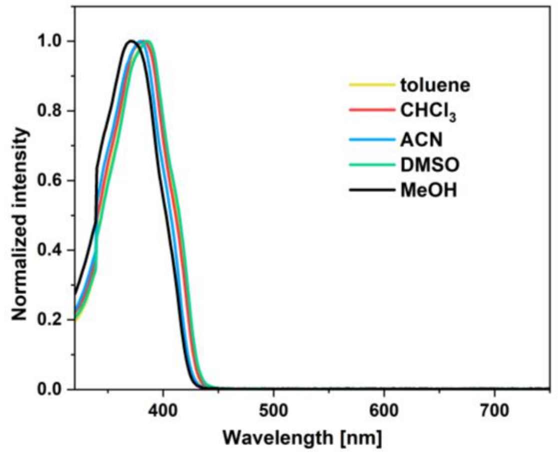

PK1 (absorption)

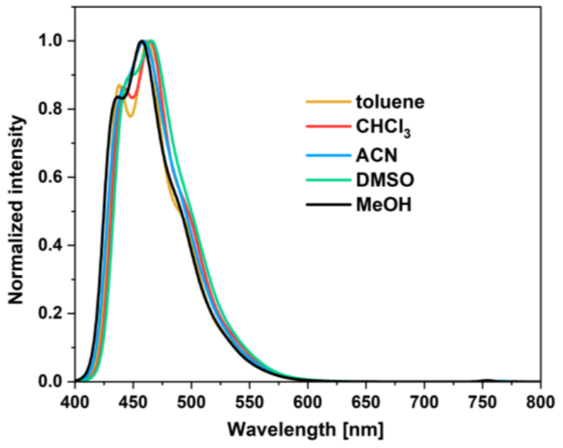

PK1 (emission)

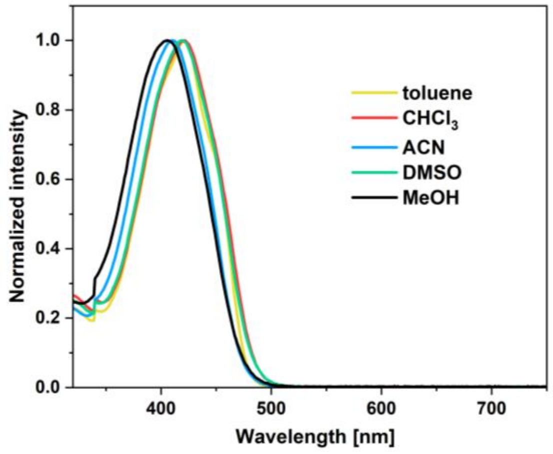

PK2 (absorption)

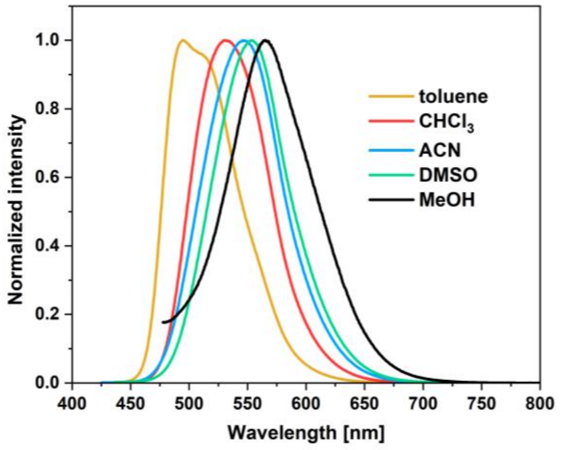

PK2 (emission)

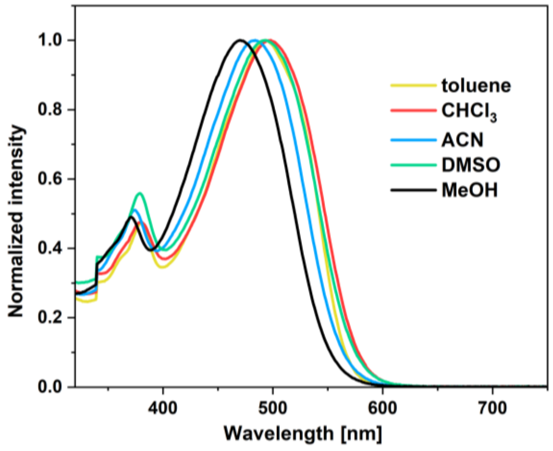

PK3 (absorption)

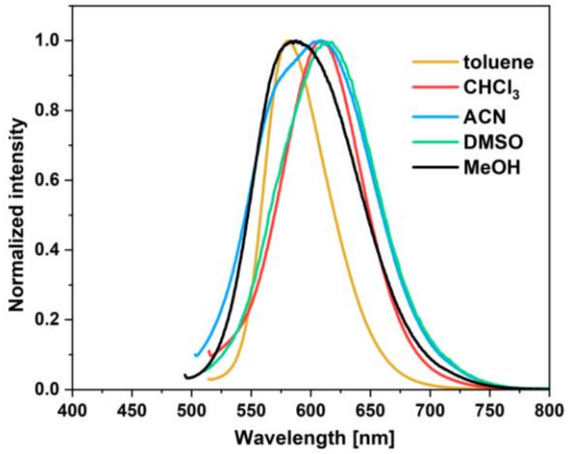

PK3 (emission)

Fig. S11. Comparison of absorption and emission behaviour of **PK** series.

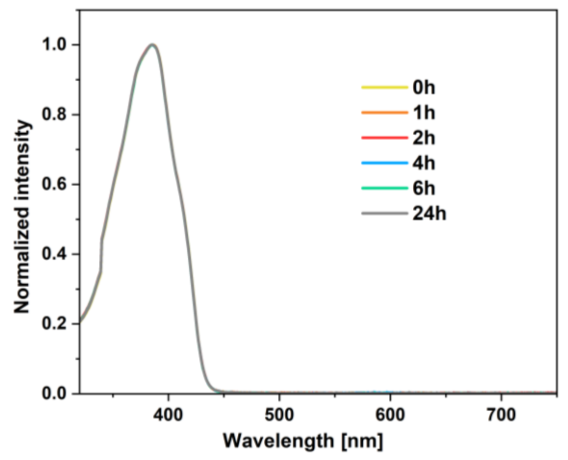

PK1

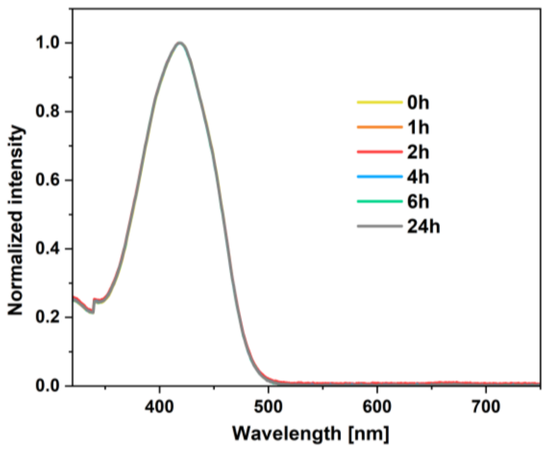

PK2

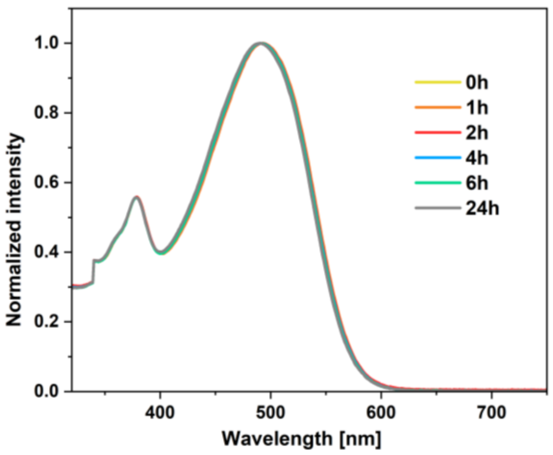

PK3

Fig. S12. UV–Vis spectra recorded in DMSO for 24 hours at room temperature.

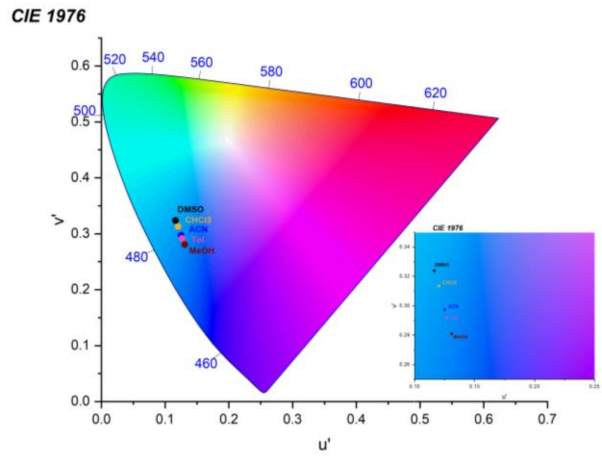

PK1

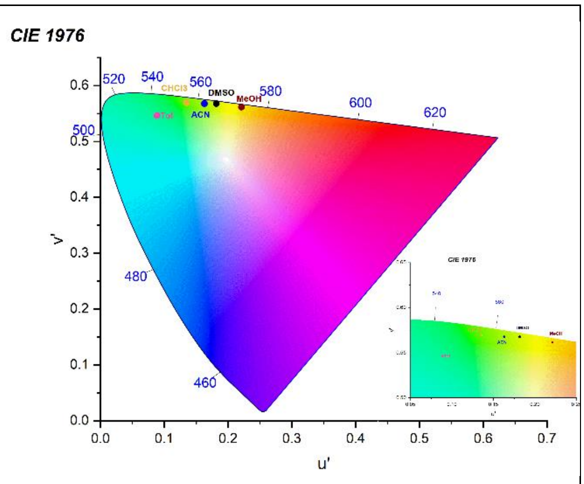

PK2

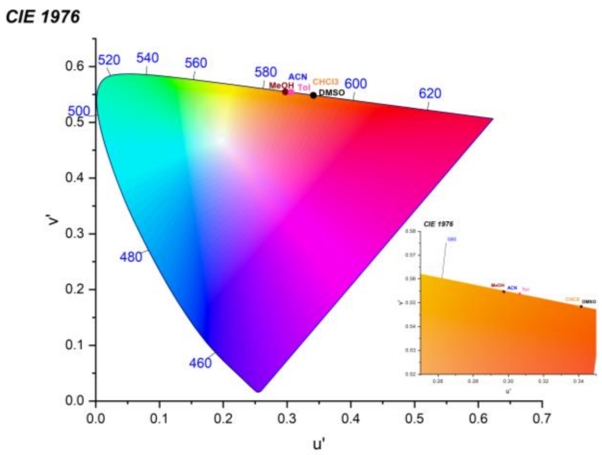

PK3

Fig. S13. Chromaticity plots of the PK series in various solvents.

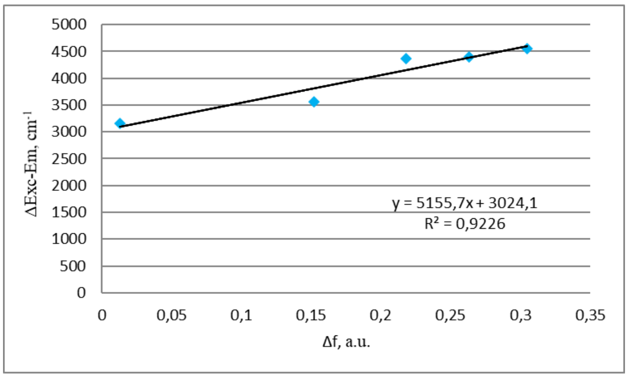

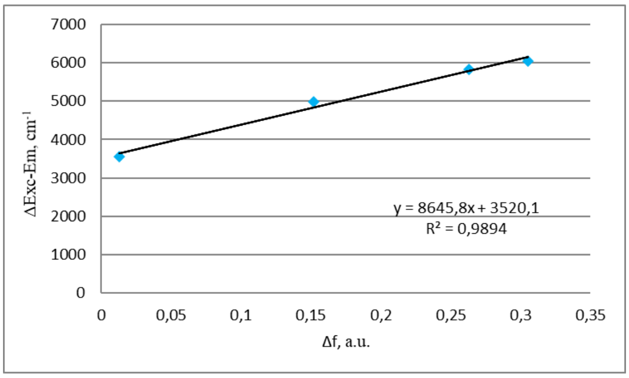

PK1	PK2

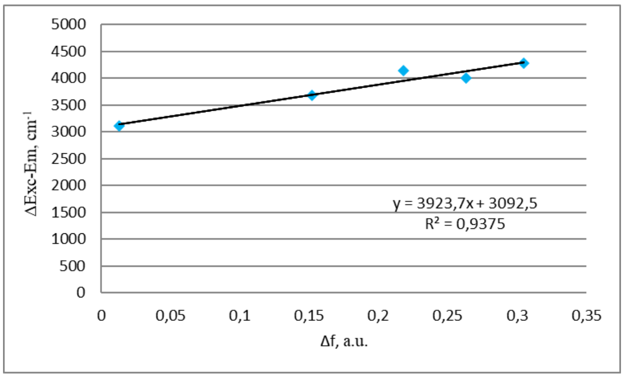

PK3

Fig. S14. The Lippert-Mataga plots

6. DFT calculations - methodology

All geometrical parameters of investigated molecules in their ground (S_{GS}) and excited (S_{CT}) states were calculated using density functional theory (DFT) approach implemented in Gaussian 16 program package [8] with PBE0/6-311++G(d,p) basis set. In order to verify that all the structures correspond to the minima on the potential energy surface, an analysis of Hessians was performed. The electronic properties were characterized by computations of the vertical absorption, and were obtained using the time-dependent density functional theory (TDDFT/PBE0) [9] and by including the state-specific (SS) corrected linear response (cLR) approach [10]. Based on previous experience with derivatives of this type [11-13], all calculations of linear and nonlinear optical properties were performed using the PBE0 functional.

The dipole moments and polarities of the charge-transfer state (CT) were evaluated by numerical differentiation of the excitation energies (*E*) in the presence of an electric field *F* of 0.001 a.u. strength:

$$\Delta\mu^{i}_{CT-GS} = \frac{E_{CT}(+F^{i})-E_{CT}(-F^{i})}{-2F^{i}} - \frac{E_{GS}(+F^{i})-E_{GS}(-F^{i})}{-2F^{i}} \quad (1)$$

where *i* stands for the Cartesian component of the dipole moment difference.

The isotropic average polarizability ($\langle\alpha\rangle$) and first-order hyperpolarizability (β) were determined based on the Gaussian 016 program and defined as:

$$\langle\alpha\rangle = \frac{\alpha_{xx}+\alpha_{yy}+\alpha_{zz}}{3} \quad (2)$$

$$\beta = \sum_{i=x,y,z} \frac{\mu_i\beta_i}{|\mu|} \quad (3)$$

where $\beta_i (i = x, y, z)$ is given by $\beta_i = \left(\frac{1}{3}\right)\sum_{j=x,y,z}(\beta_{ijj} + \beta_{jij} + \beta_{jji})$

The density differences were obtained at the PBE0/6-311++G(d,p) level and are represented with a contour threshold of 0.02 a.u. In these graphs, the blue (purple) zones indicate density decrease (increase) upon electronic transition. The charge transfer parameters, namely the charge-transfer distance (D_{CT}) and the amount of transferred charge (q_{CT}), have been determined following a Le Bahers' procedure [14].

The solvent effect on the linear and nonlinear optical properties has been taken into account using the Integral Equation Formalism for the Polarizable Continuum Model (IEF–PCM) [15,16].

The binding properties of considered dyes were studied by performing a series of AutoDock 4.2 [17-19] simulations. The starting structures of each complex comprised Human

Serum Albuminum (HAS) taken from PDB ID [20] and Concanavalin A (ConA) taken from PDB ID: 2a7a [21] and each dye as a ligand. The docking region on the target protein was defined by establishing a cubic grid box with the dimensions of 16Å and a grid spacing of 1Å. In AutoDock 4.2 simulations, the Lamarkian genetic algorithm was employed to identify the appropriate binding energy and conformation of compounds. For each atom of the receptor molecule, Gasteiger charges were calculated. The investigation of the binding site was performed using a united-atom scoring function implemented in AutoDock Vina [22]. The docking procedure was repeated ten times for each lysine and this enabled for identification of the sites with the highest affinity of fluorescent probes.

The biological activities were simulated using a combination of the 3D/4D QSAR BiS/MC and CoCon algorithms [23-25].

Figure S15. HOMO / LUMO plots.

PK1 **PK2** **PK3**

LUMO

HOMO

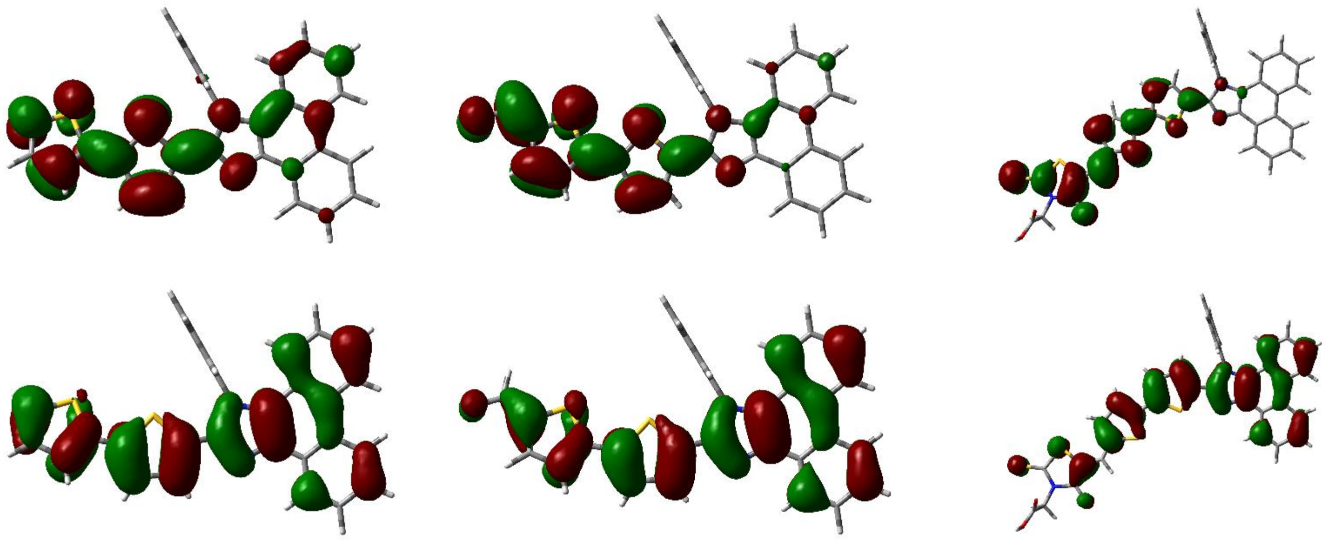

Figure S16. The MEP surfaces

PK1 **PK2** **PK3**

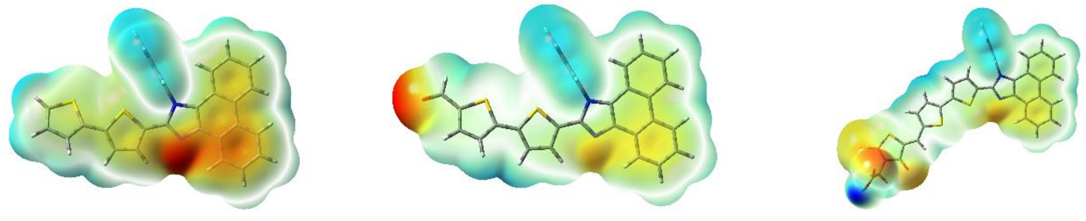

Figure S17. Density difference plots

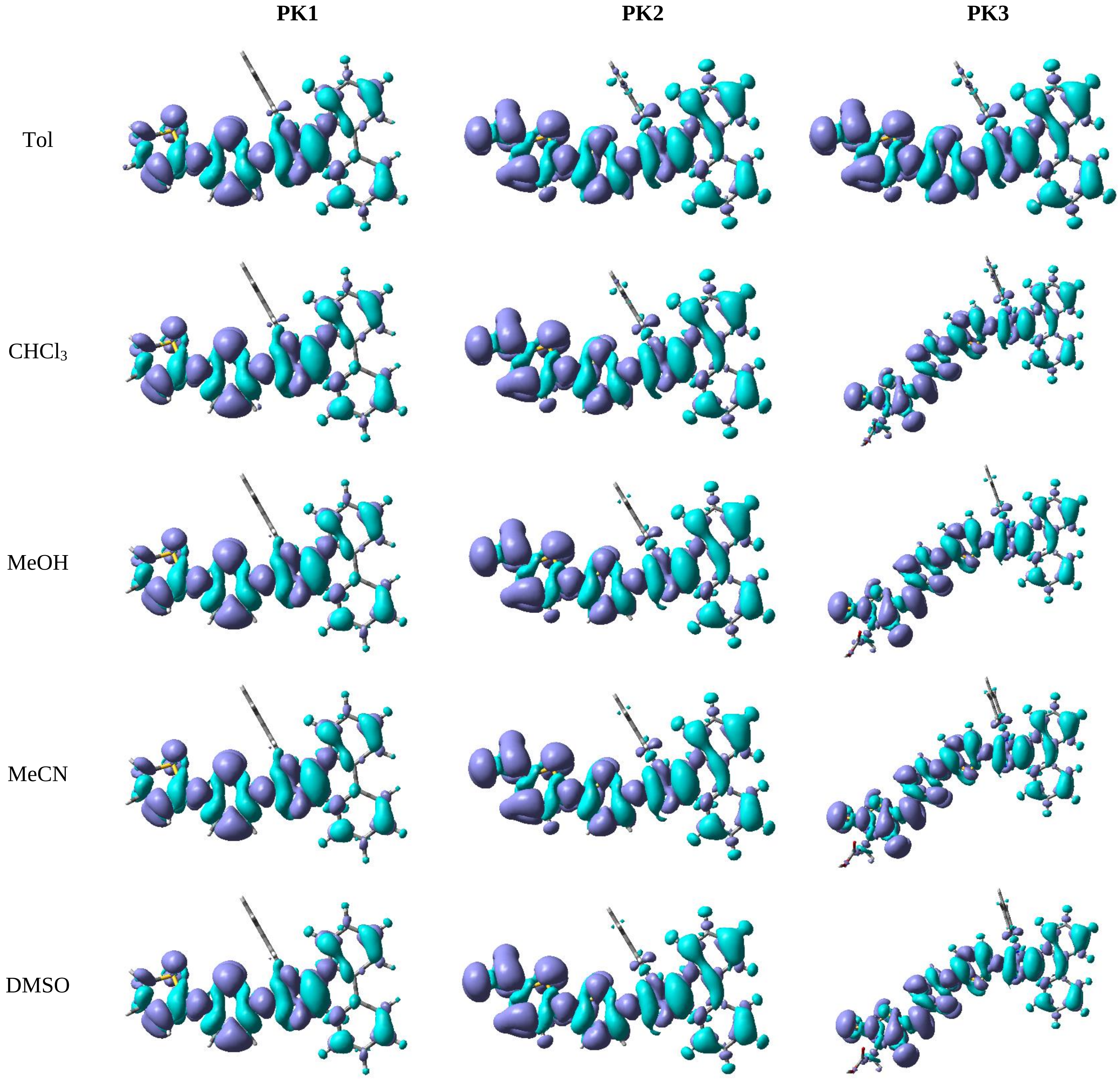

Table S1. The frontier orbital energies in tested solvents for ***Z***-isomers. Values are given in [eV]

	E_{HOMO}	E_{LUMO}	E_{GAP}	η	μ	χ	σ	π	S	ω

	Tol	-5.5380	-1.8304	3.7076	1.8538	-3.6842	3.6842	0.5394	-3.6842	0.9269	3.6610
	$CHCl_3$	-5.6232	-1.9148	3.7084	1.8542	-3.7690	3.7690	0.5393	-3.7690	0.9271	3.8305
**PK1**	MeOH	-5.6697	-1.9599	3.7098	1.8549	-3.8148	3.8148	0.5391	-3.8148	0.9274	3.9229
	MeCN	-5.6711	-1.9613	3.7098	1.8549	-3.8162	3.8162	0.5391	-3.8162	0.9274	3.9257
	DMSO	-5.6746	-1.9648	3.7098	1.8549	-3.8197	3.8197	0.5391	-3.8197	0.9274	3.9329
	Tol	-5.7111	-2.5415	3.1696	1.5848	-4.1263	4.1263	-0.6310	-4.1263	-0.7924	-5.3718
	$CHCl_3$	-5.7571	-2.5951	3.1620	1.5810	-4.1761	4.1761	0.6325	-4.1761	0.7905	5.5155
**PK2**	MeOH	-5.7838	-2.6193	3.1644	1.5822	-4.2015	4.2015	0.6320	-4.2015	0.7911	5.5786
	MeCN	-5.7846	-2.6201	3.1644	1.5822	-4.2024	4.2024	0.6320	-4.2024	0.7911	5.5807
	DMSO	-5.7868	-2.6218	3.1650	1.5825	-4.2043	4.2043	0.6319	-4.2043	0.7912	5.5848
	Tol	-5.6727	-2.9903	2.6825	1.3412	-4.3315	4.3315	0.7456	-4.3315	0.6706	6.9942
	$CHCl_3$	-5.7152	-3.0558	2.6593	1.3297	-4.3855	4.855	0.7521	-4.3855	0.6648	7.2321
**PK3**	MeOH	-5.7408	-3.0896	2.6512	1.3256	-4.4152	4.4152	0.7544	-4.4152	0.6628	7.3529
	MeCN	-5.7416	-3.0907	2.6509	1.3255	-4.4161	4.4161	0.7545	-4.4161	0.6627	7.3568
	DMSO	-5.7435	-3.0931	2.6504	1.3252	-4.4183	4.4183	0.7546	-4.4183	0.6626	7.3656

Table S2. Linear optical properties (nm)

	λ_{ABS}	f	λ_{cLR}	λ_{FL}
**PK1**				
Tol	390.94	1.1971	394.03	463.76
$CHCl_3$	390.16	1.1933	393.84	466.77
MeOH	387.79	1.1650	393.12	465.67
MeCN	388.12	1.1706	393.20	466.37
DMSO	389.60	1.1948	393.59	465.74
**PK2**				
Tol	423.83	1.1123	429.33	517.64
$CHCl_3$	423.80	1.1167	431.22	532.97
MeOH	420.30	1.1117	428.95	550.57
MeCN	420.71	1.1041	429.60	549.45
DMSO	422.58	1.1286	432.40	555.84
**PK3**				
Tol	495.99	1.5291	498.66	586.01
$CHCl_3$	498.19	1.5198	504.62	613.26

MeOH	497.06	1.5109	504.94	590.54
MeCN	497.73	1.5177	505.95	611.92
DMSO	500.62	1.5461	510.10	617.74

Table S3. Calculated values of dipole moments (in D) for the ground and CT excited state

	PK1		PK2		PK3	
	μ_{GS}	μ_{CT}	μ_{GS}	μ_{CT}	μ_{GS}	μ_{CT}
Tol	4.39	12.70	4.43	19.83	8.64	25.93
$CHCl_3$	4.75	14.22	4.71	20.89	9.35	27.50
MeOH	5.17	15.71	5.06	22.05	10.22	28.50
MeCN	5.18	15.74	5.07	22.65	10.23	28.65
DMSO	5.20	15.82	5.08	22.81	10.27	28.78

Table S4. CT parameters for the bright low-lying excited state. Values q_{CT} are given in [e] and D_{CT} in [Å]

	PK1		PK2		PK3	
	q_{CT}	D_{CT}	q_{CT}	D_{CT}	q_{CT}	D_{CT}
Tol	0.492	2.962	0.692	4.915	0.731	5.989
$CHCl_3$	0.481	3.027	0.681	5.265	0.728	6.379
MeOH	0.468	3.079	0.675	5.628	0.724	6.736
MeCN	0.467	3.072	0.674	5.628	0.724	6.728
DMSO	0.466	3.043	0.673	5.619	0.722	6.693

Table S5. Nonlinear properties. Values are given in (a.u.)

	PK1		PK2		PK3	
	$\langle\alpha\rangle$	β	$\langle\alpha\rangle$	β	$\langle\alpha\rangle$	β
Tol	525.18	1791.93	582.21	12902.12	848.71	38486.57
$CHCl_3$	571.81	1945.35	635.59	15737.35	922.44	48163.66
MeOH	631.38	2033.57	702.05	18532.92	1015.42	59988.75
MeCN	632.48	2034.26	703.25	18575.16	1017.13	60197.98

DMSO	635.28	2036.12	706.34	18680.81	1021.51	60735.83

Table S6. Two-photon absorption cross-section. Values $\langle \delta^{OF} \rangle$ are given in [a.u.] and $\sigma_{OF}^{(2)}$ in [GM]

	**PK1**		**PK2**		**PK3**	
	$\langle \delta^{OF} \rangle$	$\sigma_{OF}^{(2)}$	$\langle \delta^{OF} \rangle$	$\sigma_{OF}^{(2)}$	$\langle \delta^{OF} \rangle$	$\sigma_{OF}^{(2)}$
Tol	3078.80	10.61	25688.21	72.30	70040.50	151.76
$CHCl_3$	3117.23	10.74	26632.15	74.47	75801.71	163.04
MeOH	3155.88	10.87	27682.11	77.41	81057.50	173.06
MeCN	3155.88	10.87	27696.95	77.45	81138.64	173.23
DMSO	3155.88	10.87	27742.75	77.58	81351.94	173.68

Table S7. Free energies of solvation (kcal/mol)

	**PK1**	**PK2**	**PK3**
		ΔG_{solv}	
Tol	-22.65	-26.67	-30.57
$CHCl_3$	-25.91	-27.69	-35.27
MeOH	-24.42	-24.44	-32.89
MeCN	-25.93	-28.21	-38.58
DMSO	-19.84	-22.540	-32.41

Table S8. Biological activities (P – Probability; Pred - Prediction ; A – active; InA – inactive)

		PK1		PK2		PK3	
**Classification**	**Target**	**Pre**	**P**	**Pre**	**P**	**Pre**	**P**
Tox21-Nuclear receptor signalling pathways	Aryl hydrocarbon Receptor (AhR)	A	0.55	A	0,56	InA	0,78
Tox21-Nuclear receptor signalling pathways	Androgen Receptor (AR)	InA	0.97	InA	0,97	InA	0,96
Tox21-Nuclear receptor signalling pathways	Androgen Receptor Ligand Binding Domain (AR-LBD)	InA	0.95	InA	0,97	InA	0,96
Tox21-Nuclear receptor signalling pathways	Aromatase	InA	0.77	InA	0,73	InA	0,84
Tox21-Nuclear receptor signalling pathways	Estrogen Receptor Alpha (ER)	InA	0.75	InA	0,68	InA	0,84
Tox21-Nuclear receptor signalling pathways	Estrogen Receptor Ligand Binding Domain (ER-LBD)	InA	0.96	InA	0,96	InA	0,98
Tox21-Nuclear receptor signalling pathways	Peroxisome Proliferator Activated Receptor Gamma (PPAR-Gamma)	InA	0.96	InA	0,93	InA	0,63
Tox21-Stress response pathways	Nuclear factor (erythroid-derived 2)-like 2/antioxidant responsive element (nrf2/ARE)	InA	0.73	InA	0,80	InA	0,89
Tox21-Stress response pathways	Heat shock factor response element (HSE)	InA	0.73	InA	0,80	InA	0,89
Tox21-Stress response pathways	Mitochondrial Membrane Potential (MMP)	InA	0.57	InA	0,61	InA	0,76
Tox21-Stress response pathways	Phosphoprotein (Tumor Supressor) p53	InA	0.78	InA	0,76	InA	0,86
Tox21-Stress response pathways	ATPase family AAA domain-containing protein 5 (ATAD5)	InA	0.64	InA	0,67	InA	0,77
Molecular Initiating Events	Thyroid hormone receptor alpha (THRα)	InA	0.90	InA	0,90	InA	0,9
Molecular Initiating Events	Thyroid hormone receptor beta (THRβ)	InA	0.78	InA	0,78	InA	0,78
Molecular Initiating Events	Transtyretrin (TTR)	InA	0.97	InA	0,97	InA	0,97
Molecular Initiating Events	Ryanodine receptor (RYR)	InA	0.98	InA	0,98	InA	0,98
Molecular Initiating Events	GABA receptor (GABAR)	InA	0.96	InA	0,96	InA	0,96
Molecular Initiating Events	Glutamate N-methyl-D-aspartate receptor (NMDAR)	InA	0.92	InA	0,92	InA	0,92
Molecular Initiating Events	alpha-amino-3-hydroxy-5-methyl-4-isoxazolepropionate receptor (AMPAR)	InA	0.97	InA	0,97	InA	0,97
Molecular Initiating Events	Kainate receptor (KAR)	InA	0.99	InA	0,99	InA	0,99
Molecular Initiating Events	Achetylcholinesterase (AChE)	InA	0.53	A	0,53	InA	0,54
Molecular Initiating Events	Constitutive androstane receptor (CAR)	InA	0.98	InA	0,98	InA	0,98
Molecular Initiating Events	Pregnane X receptor (PXR)	InA	0.92	InA	0,92	InA	0,92
Molecular Initiating Events	NADH-quinone oxidoreductase (NADHOX)	InA	0.97	InA	0,97	InA	0,97
Molecular Initiating Events	Voltage gated sodium channel (VGSC)	InA	0.95	InA	0,95	InA	0,95
Molecular Initiating Events	Na+/I- symporter (NIS)	InA	0.98	InA	0,98	InA	0,98

7. Literature

[1] European Committee for Antimicrobial Susceptibility Testing (EUCAST) (2003) determination of minimum inhibitory concentrations (MICs) of antibacterial agents by broth dilution. EUCAST discussion document E. Dis 5.1, Clin. Microbiol. Infect., 9 (2003) 1-7.

[2] Clinical and Laboratory Standards Institute. Reference method for broth dilution antifungal susceptibility testing of yeasts. M27-S4. Clinical and Laboratory Standards Institute, Wayne, PA, USA, (2012).

[3] A. Biernasiuk; M. Kawczyńska; A. Berecka-Rycerz; B. Rosada; A. Gumieniczek; A. Malm; K. Dzitko; K.Z. Łączkowski: Synthesis, antimicrobial activity, and determination of the lipophilicity of ((cyclohex-3-enylmethylene)hydrazinyl)thiazole derivatives. Med. Chem. Res. 28 (2019) 2023-2036.

[4] A. Szlapa-Kula; S. Kula; Ł. Kaźmierski; A. Biernasiuk; P. Krawczyk, Can a Small Change in the Heterocyclic Substituent Significantly Impact the Physicochemical and Biological Properties of (*Z*)-2-(5-Benzylidene-4-oxo-2-thioxothiazolidin-3-yl)acetic Acid Derivatives? Sensors 24 (2024) 1524-1543.

[5] I. Wiegand; K. Hilpert; R.E.W. Hancock: Agar and broth dilution methods to determine the minimal inhibitory concentration (MIC) of antimicrobial substances. Nat. Protoc., 3 (2008) 163-75.

[6] F. O'Donnell; T.J. Smyth; V.N. Ramachandran; W.F. Smyth: A study of the antimicrobial activity of selected synthetic and naturally occurring quinolines. Int. J. Antimicrob. Agents., 35 (2010) 30-38.

[7] S. Kula, A. Szlapa-Kula, S. Kotowicz, M. Filapek, K. Bujak, M. Siwy, H. Janeczek, S. Maćkowski, E. Schab-Balcerzak, Dyes and Pigments, 159 (2018) 646-654.

[8] M.J. Frisch, G.W. Trucks, G.B. Schlegel et al. Gaussian 09, Revision A.1, Gaussian, Inc., Wallingford CT, (2009).

[9] C. Adamo, G.E. Scuseria, V. Barone, Accurate excitation energies from time-dependent density functional theory: Assessing the PBE0 model, J. Chem. Phys. 111 (1999) 2889-2899. DOI: 10.1063/1.479571

[10] C. Guido, S. Caprasecca, How to perform corrected Linear Response calculations in G09. https://www1.dcci.unipi.it/molecolab/tools/white-papers/pisalr/; (2016). DOI: 10.13140/RG.2.1.1903.7845

[11] S. Kula, Ł. Kaźmierski, M. Filapek, P. Krawczyk. Influence of a bithiophene substituent on the properties of a new fluorescent probe – theoretical and experimental study. J. Mol. Struct. 1281 (2023) 135151. DOI:10.1016/j.molstruc.2023.135151

[12] S. Kula, P. Krawczyk, Ł. Kaźmierski, M. Filapek. Synthesis, physicochemical characterization and biological properties of new 5-(1*H*-phenanthro[9,10-d]imidazol-2-yl)-thiophene-2-carbaldehyde. J. Mol. Struct. 1252 (2022) 1–13. https://doi.org/10.1016/j.molstruc.2021.132122

[13] S. Kula, P. Krawczyk, M. Filapek, A. M. Maroń. Influence of N-donor substituents on physicochemical properties of phenanthro[9,10-d]imidazole derivatives. J. Lumin. 233 (2021) 117910. https://doi.org/10.1016/j.jlumin.2021.117910

[14] M. Caricato, A comparison between state-specific and linear-response formalisms for the calculation of vertical electronic transition energy in solution with the CCSD-PCM method, M. J. Chem. Phys. 139 (2013) 044116. DOI: 10.1063/1.4816482

[15] T. Le Bahers, C. Adamo, I. Ciofini, A Qualitative Index of Spatial Extent in Charge-Transfer Excitations, J. Chem. Theory Comput. 7 (2011) 2498–2506. DOI: 10.1021/ct200308m

[16] M.T. Cancés, B. Mennucci, J. Tomasi, A new integral equation formalism for the polarizable continuum model: Theoretical background and applications to isotropic and anisotropic dielectrics, J. Chem. Phys. 107 (1997) 3032-3041. DOI: 10.1063/1.474659

[17] G.M. Morris, R. Huey, W. Lindstrom, M.F. Sanner, R.K. Belew, D.S. Goodsell, A.J. Olson, AutoDock4 and AutoDockTools4: Automated docking with selective receptor flexibility, J. Comput. Chem. 30 (2009) 2785-2791. DOI: 10.1002/jcc.21256

[18] S. Cosconati, S. Forli, A.L. Perryman, R. Harris, D.S. Goodsell, A.J. Olson, Virtual screening with AutoDock: theory and practice, Expert Opin. Drug Discovery 5 (2010) 597-607. DOI: 10.1517/17460441.2010.484460

[19] S. Forli, A.J. Olson, A Force Field with Discrete Displaceable Waters and Desolvation Entropy for Hydrated Ligand Docking, J. Med. Chem. 55 (2012) 623-638. DOI: 10.1021/jm2005145

[20] S. Sugio, S. Mochizuki, M. Noda, A. Kashima, Crystal structure of human serum albumin, Protein Data Bank, Rutgers University (1998). https://doi.org/10.2210/pdb1ao6/pdb

[21] C. S. Panjikar, P.A. Tucker, M.S. Weiss, On the Routine Use of Soft X-Rays in Macromolecular Crystallography, Part III- The Optimal Data Collection Wavelength, Acta Crystallogr. D Biol. Crystallogr. 61 (2005) 1263-1272. DOI: 10.2210/pdb2a7e/pdb

[22] O. Trott, A.J. Olson, AutoDock Vina: Improving the speed and accuracy of docking with a new scoring function, efficient optimization, and multithreading, J. Comp. Chem. 31 (2010) 455-461. DOI: 10.1002/jcc.21334
[23] V. Potemkin, M. Grishina, Principles for 3D/4D QSAR classification of drugs, Drug Discov. Today 13 (2008) 952-959. DOI:10.1016/j.drudis.2008.07.006
[24] V. Potemkin, M. Grishina, A new paradigm for pattern recognition of drugs, **J**. Comput. Aided. Mol. Des. 22 (2008) 489-505. DOI:10.1007/s10822-008-9203-x
[25] V. Potemkin, A.A. Pogrebnoy, M.A. Grishina, Technique for Energy Decomposition in the Study of "Receptor-Ligand" Complexes, J. Chem. Inf. Model. 49 (2009) 1389-406. DOI: 10.1021/ci800405n
